# Human umbilical cord-derived mesenchymal stem cells ameliorate perioperative neurocognitive disorder by inhibiting inflammatory responses and activating BDNF/TrkB/CREB signaling pathway in aged mice

**DOI:** 10.1186/s13287-023-03499-x

**Published:** 2023-09-21

**Authors:** Penghui Wei, Min Jia, Xiangyi Kong, Wenyuan Lyu, Hao Feng, Xinyi Sun, Jianjun Li, Jian-jun Yang

**Affiliations:** 1 Department of Anesthesiology, Pain and Perioperative Medicine, The First Affiliated Hospital of Zhengzhou University, No. 1 East Jianshe Road, Zhengzhou, 450052 People’s Republic of China; 2 Department of Anesthesiology, Qilu Hospital (Qingdao), Cheeloo College of Medicine, Shandong University, Qingdao, People’s Republic of China; 3 Neuroscience Research Institute, Zhengzhou University Academy of Medical Sciences, Zhengzhou, People’s Republic of China; 4 Henan Province International Joint Laboratory of Pain, Cognition and Emotion, Zhengzhou, People’s Republic of China; 5 Department of Anesthesiology, Qilu Hospital, Cheeloo College of Medicine, Shandong University, Jinan, People’s Republic of China

**Keywords:** Human umbilical cord-derived mesenchymal stem cells, Cognitive impairment, Aging brain, Perioperative neurocognitive disorder, BDNF/TrkB, Lipid metabolism, Inflammation

## Abstract

**Background:**

Perioperative neurocognitive disorder (PND) is a key complication affecting older individuals after anesthesia and surgery. Failure to translate multiple pharmacological therapies for PND from preclinical studies to clinical settings has necessitated the exploration of novel therapeutic strategies. Human umbilical cord-derived mesenchymal stem cells (hUC-MSCs) treatment has emerged as a promising therapeutic strategy for treating neurodegenerative diseases and has the potential to translate basic science into clinical practice. In this study, we investigated the effects and underlying mechanism of hUC-MSCs on PND in aged mice.

**Methods:**

hUC-MSCs were isolated from an infant umbilical cord and identified using flow cytometry and differentiation assays. We established PND model by undergoing aseptic laparotomy under isoflurane anesthesia maintaining spontaneous ventilation in eighteen-month-old male C57BL/6 mice. hUC-MSCs were slowly injected into mice by coccygeal vein before anesthesia. Cognitive function, systemic and neuroinflammatory responses, neuroplasticity, endogenous neurogenesis, and brain-derived neurotrophic factor (BDNF) were assessed. To determine the brain mechanisms underlying by which hUC-MSCs mediate their neuroprotective effects in PND, K252a, an antagonist of BDNF receptor, was administered intraperitoneally before surgery. Hippocampal BDNF/TrkB/CREB signaling pathway and metabolomic signatures were evaluated.

**Results:**

hUC-MSC treatment ameliorated the learning and memory impairment in aged mice with PND. The downstream effects were the suppression of systemic and hippocampal inflammation and restoration of neurogenesis and neuroplasticity dysregulation. Interestingly, the level of mature BDNF, but not that of proBDNF, was increased in the hippocampus after hUC-MSC treatment. Further analysis revealed that the improved cognitive recovery and the restoration of neurogenesis and neuroplasticity dysregulation elicited by exposure to hUC-MSCs were, at least partially, mediated by the activation of the BDNF/TrkB/CREB signaling pathway. Untargeted metabolomic further identified lipid metabolism dysfunction as potential downstream of the BDNF/TrkB/CREB signaling pathway in hUC-MSC-mediated neuroprotection for PND.

**Conclusions:**

Our study highlights the beneficial effects of hUC-MSC treatment on PND and provides a justification to consider the potential use of hUC-MSCs in the perioperative period.

**Supplementary Information:**

The online version contains supplementary material available at 10.1186/s13287-023-03499-x.

## Background

With the expansion of the older population worldwide, the number of surgical procedures requiring anesthesia is increasing each year. While surgery can cure disease, prolong life, and improve quality of life, many older patients develop perioperative neurocognitive disorder (PND) following major surgery [1, 2]. The aging brain—with more limited cognitive reserve—is more susceptible to the stress of anesthesia and surgery and more vulnerable to cognitive impairment than the younger brain [3]. Furthermore, older patients with PND often suffer from problems with memory, attention, and concentration that can last months and even a lifetime [4]. The International Study, including patients undergoing non-cardiac surgery, found that PND was present in 9.9% of older patients three months after surgery [5]. PND also considerably increases mortality three months after surgery and is associated with a high risk of dementia and disability, leading to the loss of employment and a greater reliance of patients on social security [6]. However, the causes of PND are still not fully understood, and there are no effective pharmacologic agents used in clinical practice for the cure or prevention of PND, although many pharmacological interventions have been investigated in preclinical studies [7]. Importantly, recent failure to translate pharmacological therapies for PND from preclinical studies to clinical settings has necessitated the exploration of novel therapeutic strategies [8].

Stem-cell therapy can overcome the deficits of traditional pharmacological therapies. It is being extensively explored as a novel treatment for neurodegenerative diseases because of its multi-target potential to alleviate cognitive impairment [9]. Mesenchymal stem cells (MSCs) are multipotent progenitors derived from various tissues, including the umbilical cord, bone marrow, amniotic fluid, and adipose tissue. MSCs can greatly expand while retaining their multipotent potential and are a potent cell source for stem-cell therapy [10]. Recently, MSCs have shown beneficial effects in treating different types of neurodegenerative diseases, including Alzheimer's and Parkinson’s disease [10, 11]. Compared with the adult MSCs, human umbilical cord-derived MSCs (hUC-MSCs) are safer and younger, grow more rapidly, and involve fewer ethical concerns and invasive procedures [12]. These advantages make hUC-MSCs a preferable transplantation candidate for treatment of neurodegenerative diseases.

The role of inflammatory responses in PND has been extensively studied. The innate immune system activation induced by surgery is essential in PND’s onset and progression. Tissue damage following anesthesia and surgery activates peripheral immune cells and promotes release of systemic inflammatory cytokines that can directly enter the brain parenchyma across the damaged blood–brain barrier and triggers a cascade of neuroinflammatory responses [3, 13]. Immunomodulation represents key mechanisms of action involved in the potential therapeutic activity of hUC-MSCs. In fact, hUC-MSCs have been shown to regulate systemic inflammation and neuroinflammation to improve cognition [14]. The therapeutic effects of MSCs may also be associated with activating brain-derived neurotrophic factor (BDNF) signaling pathway and improving synaptic function and neurogenesis [11, 12, 15]. Interestingly, reduction in hippocampal BDNF and impairments in synaptic function and endogenous neurogenesis have been implicated in the development of PND [16–18]. However, experimental study is yet to support the clinical application of hUC-MSCs in PND patients. Therefore, an animal model of PND was established by performing exploratory laparotomy under isoflurane anesthesia, a routine major surgical procedure in older adults, in aged mice. We then explored the therapeutic effects of hUC-MSCs on PND and determined the potential mechanisms through which hUC-MSCs mediated neuroprotection.

## Methods

### Animals and housing conditions

Eighteen-month-old male C57BL/6 mice weighing 28–32 g were obtained from the Jinan Pengyue Animal Company. All animals were housed (five mice per cage) in an air-conditioned room kept at 21 ± 2 °C with a relative humidity of 45 ± 5% and 12-h light/dark cycle. They were fed standard rodent food and water ad libitum and acclimated to the environment for one week before commencing the experimental procedures. All efforts were made to minimize the number and suffering of the animals used in the study. A total of 200 aged mice were used. The number of mice that contributed data for analysis in each experiment was indicated in figure legends. All animal experiments adhere to the ARRIVE guidelines. The experimental protocol was approved by the Animal Care and Use Committee of Qilu Hospital (Qingdao), Shandong University (approval no: KYDWLL-202221), and was conducted in accordance with the guidelines for experimental animal use established by the Institutional Animal Care and Use Committee of Shandong University.

### hUC-MSC preparation, characterization and differentiation

#### hUC-MSC preparation

An umbilical cord for MSC isolation and culture was obtained at Qilu hospital from a full-term healthy infant delivered after a cesarean section, whose mother had signed an informed consent form and the procedure was approved by the Qilu hospital’s human research ethics committee (approval no: KYLL-2021(KS)-086). The isolation and culture of the hUC-MSCs were conducted according to protocols published by our group and previously described procedures [19, 20]. The isolated hUC-MSCs were cultured at 37 °C with a humidified atmosphere with 5% CO_2_. The medium was changed every 3–4 days. After reaching 90% confluence, the cells were trypsinized using 0.25% trypsin and passaged at 1 × 10^4^ cells/cm^2^ to passage 5 used in the study.

### hUC-MSC characterization

The characteristics of hUC-MSCs were evaluated using flow cytometry. Passage 5 hUC-MSCs were trypsinized and centrifuged. Next, 1 × 10^6^ cells were resuspended in 100 μL of cold phosphate-buffered saline (PBS) per sample and then stained with APC anti-human CD166 antibody (Biolegend, 343906), APC anti-human CD29 antibody (Biolegend, 303008), FITC anti-human CD90 (Thy1) antibody (Biolegend, 328108), FITC anti-human HLA-DR antibody (Biolegend,327006), FITC anti-human CD73 (Ecto-5’-nucleotidase) antibody (Biolegend, 344016), FITC anti-human CD105 antibody (Biolegend, 323204), FITC anti-human CD45 antibody (Biolegend, 304006), respectively. After 15 min, the cells were washed and resuspended in 100 μL PBS. The cells were analyzed using a flow cytometer (BD Biosciences, FACSCelesta™). In total, 50,000 events were collected per sample. The data were analyzed using FlowJo software (Tree Star Inc., Ashland, OR, USA).

### hUC-MSC differentiation

The hUC-MSC differentiation potentials to adipocytes, osteocytes and chondrocytes were confirmed in accordance with the manufacturer’s instructions. For these assays, the cells were cultured and induced with adipogenic differentiation medium (Oricell, HUXUC-90031), osteogenic differentiation medium (Oricell, HUXUC-90031) and chondrogenic differentiation medium (Oricell, HUXUC-90031), and stained with Oil Red O, Alizarin Red and Alcian Blue.

### PND animal model and drug administration/hUC-MSC transplantation

A modified aseptic exploratory laparotomy was performed under isoflurane anesthesia as described in our previous studies [21, 22]. Mice were induced to lose the righting reflex with 3.5% isoflurane and 100% oxygen in a chamber while maintaining spontaneous ventilation. The mice were then placed in the right lateral position and maintained with 1.5% isoflurane for 20 min. The surgical procedure was performed under 1.5–2% isoflurane and 100% oxygen using a mouse anesthesia mask. A rectal temperature of 37 °C was maintained by using a heated blanket during the procedure. Abdominal entry was performed using a 1-cm midline vertical incision after shaving and cleaning the skin with an iodophor disinfectant. The operator vigorously manipulated the viscera and musculature by inserting a sterile probe into the peritoneal cavity and stretching the musculature without damaging the intraperitoneal organs. Subsequently, a 2-cm intestine was exteriorized and vigorously rubbed using two sterile swabs for 30 s and then placed back into the peritoneal cavity. The peritoneal lining and skin were closed and sutured using 4–0 sterile surgical sutures. The wound was covered with polysporin (Johnson & Johnson Inc.) to relieve and prevent postoperative pain and infection. The procedure lasted approximately 15 min, and the total duration of anesthesia was 1.5 h. Surgery did not impair limb function, ensuring normal activities in behavioral tests.

On the day of establishment of the animal model, cultured hUC-MSCs were harvested and suspended in normal saline (0.9% NaCl). A total dose of 2.8 × 10^7^ cells/kg in 100 µL normal saline per mouse was slowly injected into mice by coccygeal vein using a 31-G needle 1 h before anesthesia. The number of hUC-MSCs used in the study was based on previous reports [23, 24] and our pilot study. The dosages were tolerated by the mice that did not cause side effects, such as pulmonary embolism. The same volume of normal saline was injected into mice in the other groups. In second experiment, K252a (an antagonist of the TrkB receptor, MCE, 99533-80-9) diluted in 0.1% methyl sulfoxide (DMSO) was intraperitoneally administered at 25 μg/kg per injection daily for 15 days before hUC-MSC transplantation. Based on previous reports, a low concentration of K252a was selected to avoid any unwanted effects [25–27]. The same amount of DMSO and normal saline were administered intraperitoneally to mice in other groups. Mice were randomly allocated into groups based on computer-generated randomization tables in each experiment.

### Behavioral tests

Cognitive function was evaluated using the novel object recognition (NOR) test for recognition memory and the Morris water maze (MWM) test for hippocampus-dependent spatial learning and memory. All behavioral tests were conducted between 9:00 am and 5:00 pm in a quiet room with dim light.

#### NOR test

The NOR test was conducted in an opaque square chamber on a white table above the ground (400 mm *L* × 400 mm *W* × 400 mm *H*). Each mouse was brought into the test room and allowed to habituate for 5 min in an open field without any objects the day before the test. The objects were placed at opposite angles to the chamber, and an adhesive was used to secure the objects in place. The mice were placed in the chamber with their backs turned toward the objects. The chambers and objects were cleaned with 70% ethanol before each trial. Training and testing during the NOR test occurred over 2 days. The training session was performed 24 h after habituation, and the mice were freely exposed to two identical objects for 10 min. One of the objects was replaced by another novel object of different colors and shapes 1 or 24 h later. The time spent within the zone for each object (“N” for the novel object and “O” for the old object) was automatically recorded by a video tracking system (Shanghai Jiliang Software Technology Co., Ltd., JLBehv-LR4) above the chamber. The NOR test was quantified as a discrimination index (N/N + O).

#### MWM test

Six days after surgery, mice were subjected to the MWM test in a quiet environment. Mice were tested in a black circular apparatus (1200 mm diameter, 500 mm depth) filled with water containing milk to a depth of 300 mm (water temperature, 22 °C). An escape platform (diameter, 100 mm) was placed in the first quadrant and submerged 15 mm below the water surface. The MWM test consists of 5 days of spatial acquisition trials, and a 1-day probe trial was performed as previously described [26, 28]. In the spatial acquisition trials, mice were trained to find the hidden platform within 60 s using distinctive distal visual cues around the apparatus over four consecutive trials conducted once per day. Once the mice failed to locate the platform within 60 s, they were guided. Each trial in a different quadrant had a fixed starting position on the circular apparatus. Mice were placed in the pool facing the wall and picked up after remaining on the platform for 30 s in each trial. In the probe trials, the platform was removed, and the mice were allowed to swim freely for 60 s using the third quadrant (reverse quadrant) as the starting position, 2 or 24 h after the spatial acquisition trials. Parameters, including escape latency (time to reach the platform), swimming speed, and number of platform crossings (original platform position), were automatically recorded by a video tracking system (Shanghai Jiliang Software Technology Co., Ltd., DigBehv-MM).

### Immunofluorescence staining

The mice were exposed to terminal isoflurane anesthesia at 24 h or 15 days after surgery. The thoracic cavity was opened to expose the heart, and the right atrium was cut. A solution of ice-cold saline, followed by 4% paraformaldehyde, was transcardially perfused. Brains were removed and post-fixed in 4% paraformaldehyde overnight at 4 °C and then dehydrated in 30% sucrose for 3–5 days until sinking to the bottom of the vial. The brains were embedded in OCT compound (Sakura, 4583) and sliced coronally into 40 µm sections on a cryostat at − 20 °C. Sections, including the hippocampus, were washed with 0.1 M PBS before immunostaining. Then, the sections were blocked using 1% bovine serum albumin for 1 h at room temperature and incubated overnight at a 4 °C with primary antibodies: rabbit anti-Iba-1 (ionized calcium binding adaptor molecule-1, 1:500, Abcam, ab178846), rabbit anti-GFAP (glial fibrillary acidic protein, 1:500, Proteintech, 16825-1-AP), mouse anti-Sox2 (1:200, Proteintech, 66411-1-lg), mouse anti-DCX (doublecortin, 1:100, Santa Cruz Biotechnology, sc-271390), rabbit anti-PSD95 (postsynaptic density protein 95, 1:200, Abcam, ab18258), rabbit anti-synaptophysin (1:1000, Abcam, ab32127), and rabbit anti-CREB (phosphor S133, 1:100, Abcam, ab32096). After washing with PBS, the sections were incubated at room temperature for 1 h with one or two of secondary antibodies: goat anti-rabbit Alexa Fluor® 647 (1:500, Abcam, ab150083), goat anti-rabbit Alexa Fluor® 488 (1:500, Abcam, ab150081), goat anti-mouse Alexa Fluor® 488 (1:500, Abcam, ab150117) or goat anti-mouse Alexa Fluor® 647 (1:500, Abcam, ab150119). Sections were incubated with 4′,6-diamidino-2-pheny-lindole (DAPI) for nuclear staining after washing in PBS. Subsequently, the sections were mounted onto slides, and fluorescent images were captured using a Leica confocal microscope (Leica Camera, STELLARIS 5). For quantitative analysis, six sections of the hippocampus from each mouse were evaluated using the ImageJ software (National Institutes of Health, Bethesda, MD, USA). All analyses were performed in a blinded manner.

### Enzyme-linked immunosorbent assay (ELISA)

The blood was collected transcardially 6 h after surgery under terminal isoflurane anesthesia, and serum was prepared as our previously described [26]. The hippocampal tissue was harvested 24 h after surgery under terminal isoflurane anesthesia and homogenized on ice using a cell lysis buffer containing protease, phosphatase inhibitors and phenylmethanesulfonyl fluoride. The serum and supernatant were collected to measure interleukin (IL)-1β and IL-6 levels by commercially available ELISA kits according to the manufacturer’s instructions. The protein concentration in the hippocampus was normalized to the total protein content and presented as pg/mg protein. The sensitivity of the assays was 8 pg/ml for IL-1β (Proteintech, KE10003) and 3.8 pg/mL for IL-6 (Proteintech, KE10007).

### Western blotting

The entire hippocampus was dissected, homogenized and lysed in ice-cold RIPA buffer (50 mM Tris–HCl, [pH 7.5], 150 mM NaCl, 1% NP-40, 0.5% sodium deoxycholate, and 0.1% SDS) containing protease inhibitors. The samples were centrifuged at 12,000 rpm for 20 min at 4 °C to eliminate cellular debris. The supernatants were collected, and protein concentrations were measured using a BCA Protein Assay Kit (CWBIO, CW0014). Equal amounts of protein (40 μg) from each sample were loaded per lane, separated by SDS-PAGE gels and transferred onto polyvinylidene fluoride membranes. The membranes were blocked with 5% skimmed milk in Tris-buffered saline with Tween for 1 h at room temperature followed by incubation at 4 °C overnight with primary antibodies, including rabbit anti-Sox2 (1:500, Proteintech, 11064-1-AP), rabbit anti-Nestin (1:800, Proteintech, 19483-1-AP), rabbit anti-PSD95 (1:1000, Abcam, ab18258), mouse anti-DCX (1:500, Santa Cruz biotechnology, sc-271390), rabbit anti-PSD95 (1:1000, Abcam, ab18258), rabbit anti-synaptophysin (1:20000, Abcam, ab32127), rabbit anti-TrkB (Tropomyosin receptor kinase B, 1:5000, Abcam, ab187041), rabbit anti-TrkB (phosphor Y705, 1:1000, Abcam, ab229908), rabbit anti-CREB (1:1000, Proteintech, 12208-1-AP), rabbit anti-CREB (phosphor S133, 1:2000, Abcam, ab32096), rabbit anti-β-actin (1:2000, Proteintech, 20536-1-AP), rabbit anti-α-Tubulin (1:5000, Proteintech, 11224-1-AP) and rabbit anti-BDNF (1:1000, Abcam, ab108319). The BDNF antibody recognizes several BDNF isoforms, including proBDNF (28–45 kDa) and mature BDNF (14 kDa). The following day, the membranes were washed and incubated at room temperature for 1 h with goat anti-rabbit (1:3000, Proteintech, SA10000-2) or goat anti-mouse (1:5000, Proteintech, SA00001-1) HRP-conjugated secondary antibodies. Protein bands were detected using an enhanced chemiluminescence detection kit (Proteintech, PK10003) and analyzed using the ImageJ software (National Institutes of Health, Bethesda, MD, USA).

### Golgi–Cox staining

Golgi–Cox staining was performed using a Golgi Stain Kit (FD NeuroTechnologies, PK401) to examine dendritic spines density. The mice were euthanized under terminal isoflurane anesthesia, and their brains were immediately removed without perfusion and rinsed with double-distilled water to remove blood from the surface. Next, the brains were immersed in an impregnation solution, a mixture of solutions A and B (1:1), for 2–3 weeks at room temperature, away from light, and then transferred to solution C for a week. A series of 100-μm-thick coronal sections from the hippocampus were sliced using a cryostat (Leica, Wetzlar) stained and mounted onto gelatin-coated slides and then cleared in xylene and cover-slipped after alcohol dehydration. Images of the neurons in the hippocampal Cornu Ammonis 1 (CA1) region were captured using an EVOS FL automated microscope. The density of dendritic spines was detected using a confocal microscope (× 100 oil objective) and analyzed using the ImageJ software (National Institutes of Health, Bethesda, MD, USA). At least three pyramidal neurons in the CA1 region were randomly selected from each mouse, and the spine numbers were counted in a blinded manner.

### Sample preparation and untargeted metabolomic experiments

#### Sample preparation

The hippocampal tissue was harvested and weighed from MSC plus surgery group and MSC plus K252a plus surgery group at 15 days after surgery. Dried lyophilized were ground in a 2-mL Eppendorf tube, which contains a 5 mm tungsten bead for 1 min at 65 Hz in a grinding mill. Metabolites were extracted using 1-mL precooled mixtures containing methanol, acetonitrile and water (v/v/v, 2:2:1). After being placed for 1 h ultrasonic shaking in ice baths, the mixture was placed at − 20 °C for 1 h and centrifuged at 14,000×*g* for 20 min at 4 °C. Subsequently, the supernatants were recovered and concentrated to dryness in vacuum.

### UHPLC–MS/MS analysis

Metabolomics profiling analysis was performed using a UPLC-ESI-Q-Orbitrap-MS system (UHPLC, Shimadzu Nexera X2 LC-30AD, Shimadzu, Japan) coupled with Q-Exactive Plus (Thermo Scientific, San Jose, USA). For liquid chromatography (LC) separation, the hippocampal tissue was analyzed using a ACQUITY UPLC® HSS T3 column (2.1 × 100 mm, 1.8 μm) (Waters, Milford, MA, USA). The mobile phase consisted of A: 0.1% FA in water and B: 100% acetonitrile (ACN), and the flow rate was set as 0.3 mL/min. The gradient was 0% buffer B for 2 min, linearly increase to 48% in 4 min, then up to 100% in 4 min and maintained for 2 min, which was subsequently decreased to 0% buffer B in 0.1 min with 3 min re-equilibration period employed. The electrospray ionization (ESI) of positive/negative mode was separately used for MS data acquisition. The HESI source conditions included as following: spray voltage: 3.8kv (positive) and 3.2kv (negative); capillary temperature: 32 °C; sheath gas (nitrogen) flow: 30 arb (arbitrary units); Aux Gas flow: 5 arb; probe heater temp: 350 °C; S-Lens RF Level: 50. The instrument was set to acquire over the m/z range 70–1050 Da for full MS, and the full MS scans were acquired at a resolution of 70,000 at m/z 200 and 17,500 at m/z 200 for MS/MS scan. The maximum time of injection was set as 100 ms for MS and 50 ms for MS/MS, and the isolation window for MS2 was set as 2 m/z. The normalized collision energy (stepped) was set to 20, 30 and 40 for fragmentation.

### Statistical analysis

For animal experiments, statistical analyses were performed using Statistical Program for Social Sciences software (SPSS Inc., version 20.0). Graphs were drawn using GraphPad Prism (GraphPad Software Inc., version 8.0). All data are presented as the means ± standard error of the mean (SEM). Two-way repeated-measures analysis of variance (ANOVA) was used to analyze the data obtained from the spatial acquisition trials, including escape latency and swimming speed, followed by Tukey’s test. Other data were analyzed using one-way ANOVA, followed by post hoc Tukey’s multiple comparisons. A value of *P* less than 0.05 was considered statistically significant. The quantitative analyses were performed by an observer who was blind to group assignment.

For the metabolomic analysis, the raw MS data including peak alignment, retention time correction and peak area extraction were processed using MS-DIAL. The hippocampal metabolites were identified by accuracy mass (mass tolerance < 10 ppm) and MS/MS data (mass tolerance < 0.02 Da). They were matched with MassBank, HMDB, and other public databases. We only retained the variables, which had more than 50% of the nonzero measurement values in at least one group in the extracted-ion features.

## Results

### Morphologic and immunophenotypic characterization of hUC-MSCs and their differentiation capacities

**Fig. 1:**
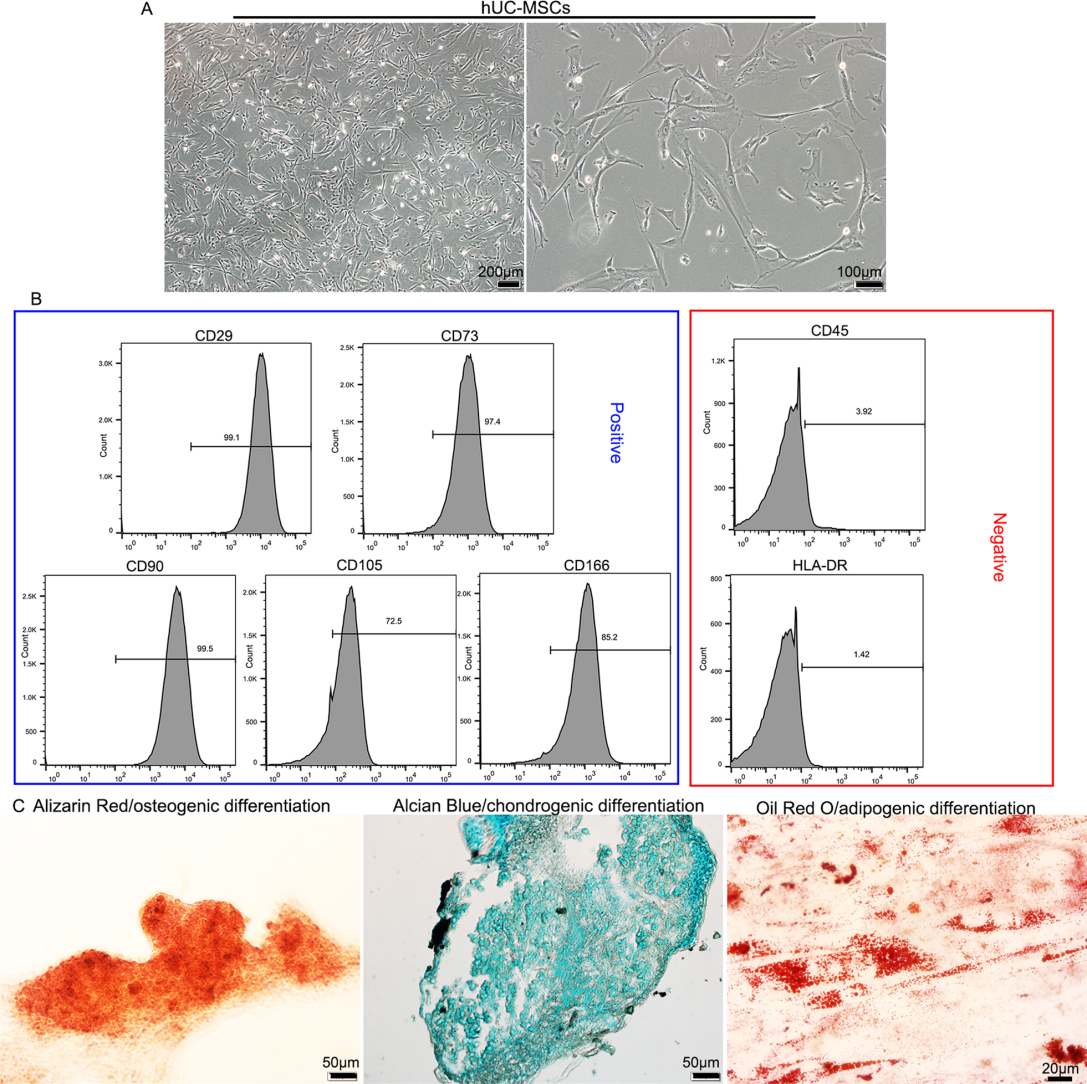
Morphology, immunophenotype and differentiation capacities of hUC-MSCs. **A** hUC-MSCs exhibited a fibroblast-like morphology at passage 3. **B** Flow cytometric analysis showed that hUC-MSCs were positive for CD29, CD73, CD90, CD105 and CD166, but negative for CD45 and HLA-DR. **C** Differentiation capacities of hUC-MSCs into osteocytes, chondrocytes and adipocytes. Osteogenic differentiation was indicated by the formation of a mineralized matrix (shown by staining with Alizarin Red). Chondrogenic differentiation was verified by the presentence of proteoglycans stained with Alcian Blue. Adipogenic differentiation was demonstrated by the formation of fat droplets positively stained with Oil red O

Adherent cells isolated from umbilical cord tissue displayed typical fibroblastic morphology after expansion to passage 3 (Fig. 1A). Flow cytometric analysis showed that hUC-MSCs at passage 5 used in this study were positive for CD29, CD73, CD90, CD105 and CD166 (mesenchymal lineage markers), but negative for CD45 (hematopoietic lineage markers) and HLA-DR (major histocompatibility complex II) (Fig. 1B). The differentiation capacities of hUC-MSCs were then assessed under different conditions (Fig. 1C). When hUC-MSCs were exposed to osteogenic conditions, the cells increasingly congregated with increasing induction time and formed a mineralized matrix, as confirmed by Alizarin Red staining. Proteoglycans gradually formed under chondrogenic conditions and were stained with Alcian Blue. Cultured hUC-MSCs flattened and broadened after adipogenic induction, and small fat droplets appeared in the cytoplasm, as confirmed by Oil Red O staining. These results are consistent with previously reported characteristics of hUC-MSCs [28].

### hUC-MSCs alleviated learning and memory impairment in aged mice with PND

**Fig. 2:**
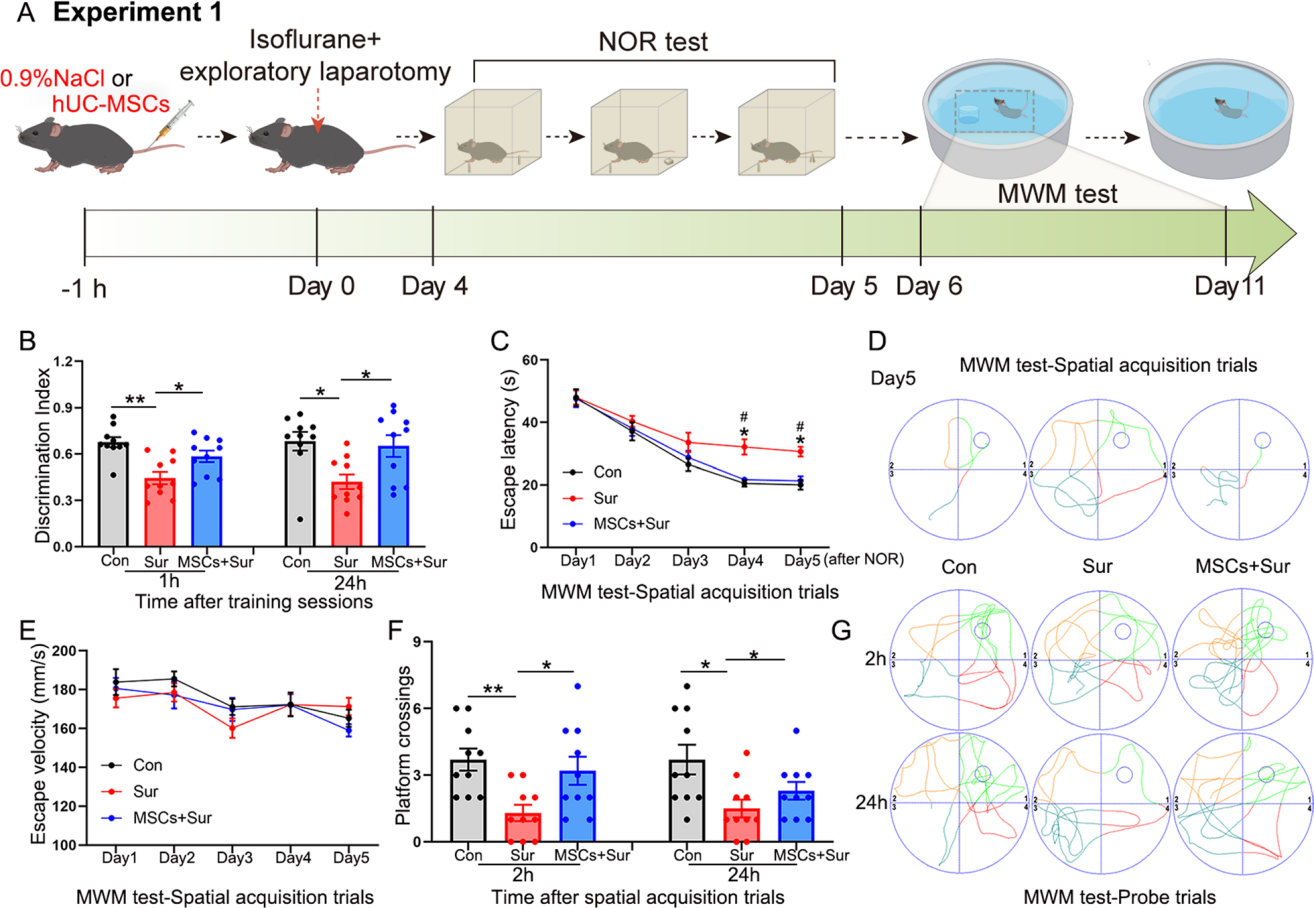
hUC-MSCs alleviated the learning and memory impairment in aged mice with PND. **A** Schematic illustrating the timeline used for hUC-MSC treatment, isoflurane anesthesia and exploratory laparotomy, novel object recognition (NOR) and Morris water maze (MWM) tests in the first experiment. **B** The NOR test was performed to measure recognition memory. **C**–**G** Hippocampus-dependent spatial learning and memory were evaluated by the MWM test consisting of spatial acquisition trials (**C**, **D** and **E**) and probe trials (**F** and **G**). **C** Escape latency to reach the platform. *P < 0.05 indicates “versus the control groups”, #*P* < 0.05 indicates “versus the MSCs plus surgery group”. **D** The typical escape routes on day 5 and **E** average swimming speed during the acquisition trials. **F** Average number of platform crossings (swims across the platform location) and **G** the typical routes within a 60-s limit in probe trials. All data are shown as mean ± S.E.M. (*n* = 10 mice). Con, control; Sur, anesthesia and surgery; hUC-MSCs, human umbilical cord-derived mesenchymal stem cells; MWM, Morris water maze; NOR, novel object recognition. *P < 0.05, **P < 0.01

hUC-MSCs were intravenously transplanted 1 h before anesthesia, and the same volume of normal saline was injected into mice in the other groups. Exploratory laparotomy was performed under isoflurane anesthesia. Mice were then tested for cognitive impairment using NOR and MWM tests from 4 to 11 days after surgery (Fig. 2A). In the NOR test, control and MSC-treated mice spent more time exploring new objects than the mice that underwent under anesthesia and surgery, either 1 h [*F*_(2, 27)_ = 10.043, *P* = 0.001] or 24 h [*F*_(2, 27)_ = 5.613, *P* = 0.009] after the initial training sessions, suggesting that control and MSC-treated mice had stronger recognition memory (Fig. 2B). Hippocampal-dependent spatial learning and memory were assessed using the MWM test. The escape latency of the control and MSC-treated mice was less than that of the mice that underwent anesthesia and surgery on days 4 [*F*_(2, 27)_ = 10.043, *P* = 0.004] and 5 [*F*_(2, 27)_ = 10.901, *P* = 0.001] (Fig. 2C). Typical escape routes on day 5 of the spatial acquisition trials are shown in Fig. 2D. The escape velocity was not significantly different between the groups, which excluded possible locomotor impairment (Fig. 2E). In the following probe trials, the hidden platform was removed, and the numbers of original platform location crossings within a 60 s were analyzed. Compared with the control and MSC-treated mice, a significant decrease in the average number of platform crossings was observed in mice that underwent anesthesia and surgery, either at 2 h [*F*_(2, 27)_ = 6.202, *P* = 0.006] or 24 h [*F*_(2, 27)_ = 5.744, *P* = 0.008] after the spatial acquisition trials (Fig. 2F). The typical routes and visible platform crossings during the probe trials are shown in Fig. 2G. These data indicate cognitive impairment after anesthesia and surgery and improvement after hUC-MSC treatment in aged mice.

### hUC-MSCs inhibited PND-related systemic and neuroinflammatory responses in aged mice

**Fig. 3:**
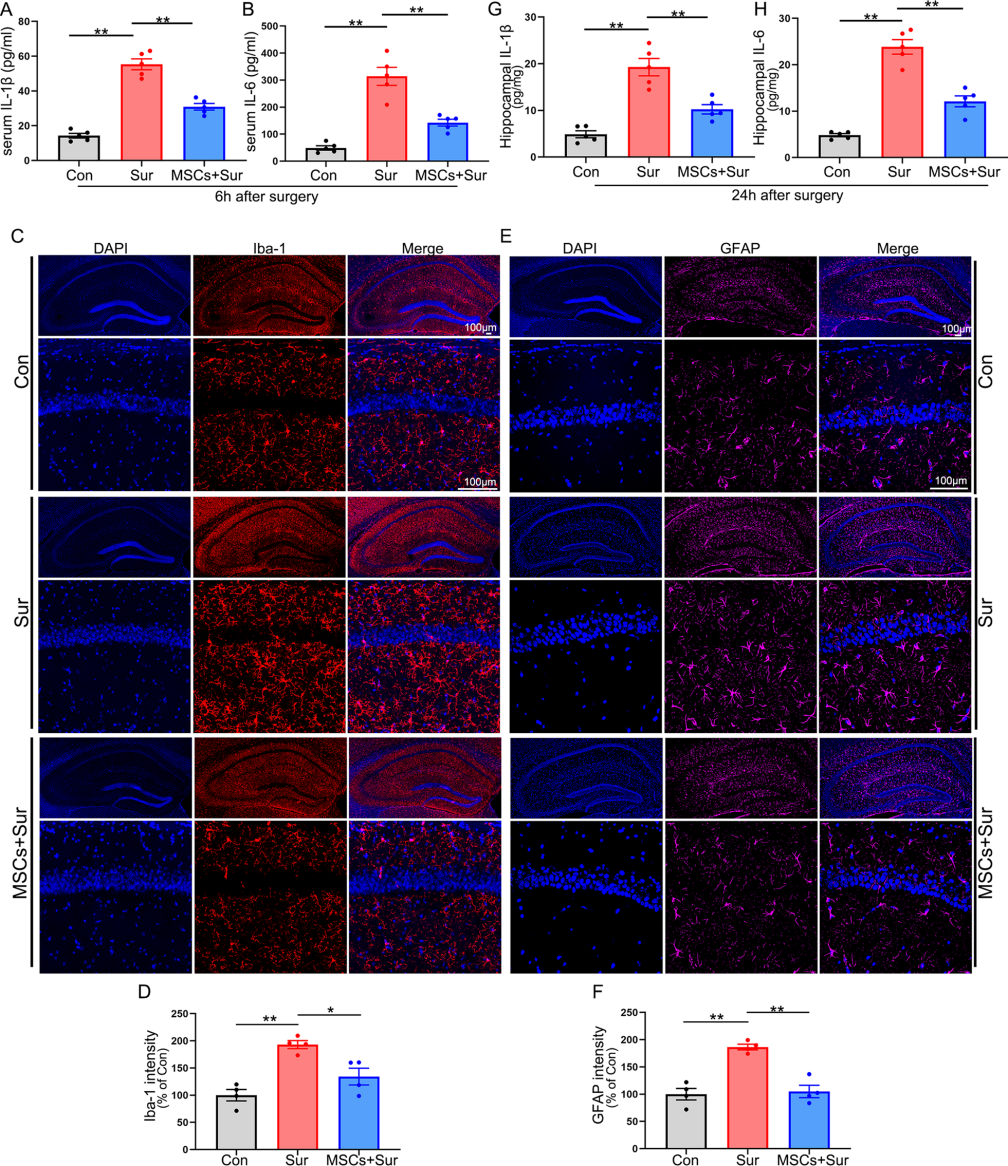
hUC-MSCs inhibited early systemic inflammatory responses to surgery and anesthesia and regulated PND-related neuroinflammatory responses in hippocampus. Blood was collected 6 h after surgery, and serum was prepared for ELISA. Hippocampus tissue was harvested 24 h after surgery for immunostaining or ELISA. Quantifications of serum IL-1β (**A**) and IL-6 (**B**) by ELISA. **C** Representative Iba-1 immunostaining images of the hippocampus. **D** Quantification of the relative fluorescent intensity for Iba-1-positive microglia in the hippocampus. **E** Representative GFAP immunostaining images of the hippocampus. **F** Quantification of the relative fluorescent intensity for GFAP-positive astrocytes in the hippocampus. Quantifications of IL-1β (**G**) and IL-6 (**H**) in the hippocampus by ELISA. All data are shown as mean ± S.E.M. (*n* = 4–5 mice). Con, control; Sur, anesthesia and surgery; hUC-MSCs, human umbilical cord-derived mesenchymal stem cells. *P < 0.05, **P < 0.01

Inflammatory responses play an important role in the pathogenesis of PND. The release of peripheral cytokines induced by surgical trauma and anesthesia is the major initiator of PND progression [3]. Six hours after surgery, there was a significant increase of both serum L-1β (Fig. 3A, [*F*_(2, 12)_ = 83.69, *P* < 0.0001]) and IL-6 (Fig. 3B, [*F*_(2, 12)_ = 40.703, *P* < 0.0001]); however, both responses were prevented by intravenous transplantation of hUC-MSCs. To determine whether hUC-MSC-mediated cognitive improvement on PND was accompanied by changes in neuroinflammatory responses in the hippocampus, we assessed Iba-1 and GFAP by immunostaining and IL-1β and IL-6 by ELISA 24 h after surgery. Microglial and astroglial activation were significantly increased in the hippocampus after anesthesia and surgery, as evidenced by the remarkable upregulation of Iba-1 [*F*_(2, 9)_ = 16.533, *P* = 0.001] and GFAP [*F*_(2, 9)_ = 26.395, *P* < 0.0001]. hUC-MSCs strongly inhibited microglial and astroglial activation (Fig. 3C–F). The levels of proinflammatory cytokines were measured 24 h after surgery. Anesthesia and surgery significantly increased IL-1β [*F*_(2, 12)_ = 31.709, *P* < 0.0001] and IL-6 [*F*_(2, 12)_ = 70.205, *P* < 0.0001] expression levels in the hippocampus and hUC-MSC treatment reversed these increases (Fig. 3G, H). These results suggest that intravenously transplanted hUC-MSCs can inhibit early systemic inflammatory responses to surgery and regulate PND-related neuroinflammatory responses in the hippocampus, which may initially block PND progression.

### hUC-MSCs restored endogenous neurogenesis and neuroplasticity dysregulation in aged mice with PND

**Fig. 4:**
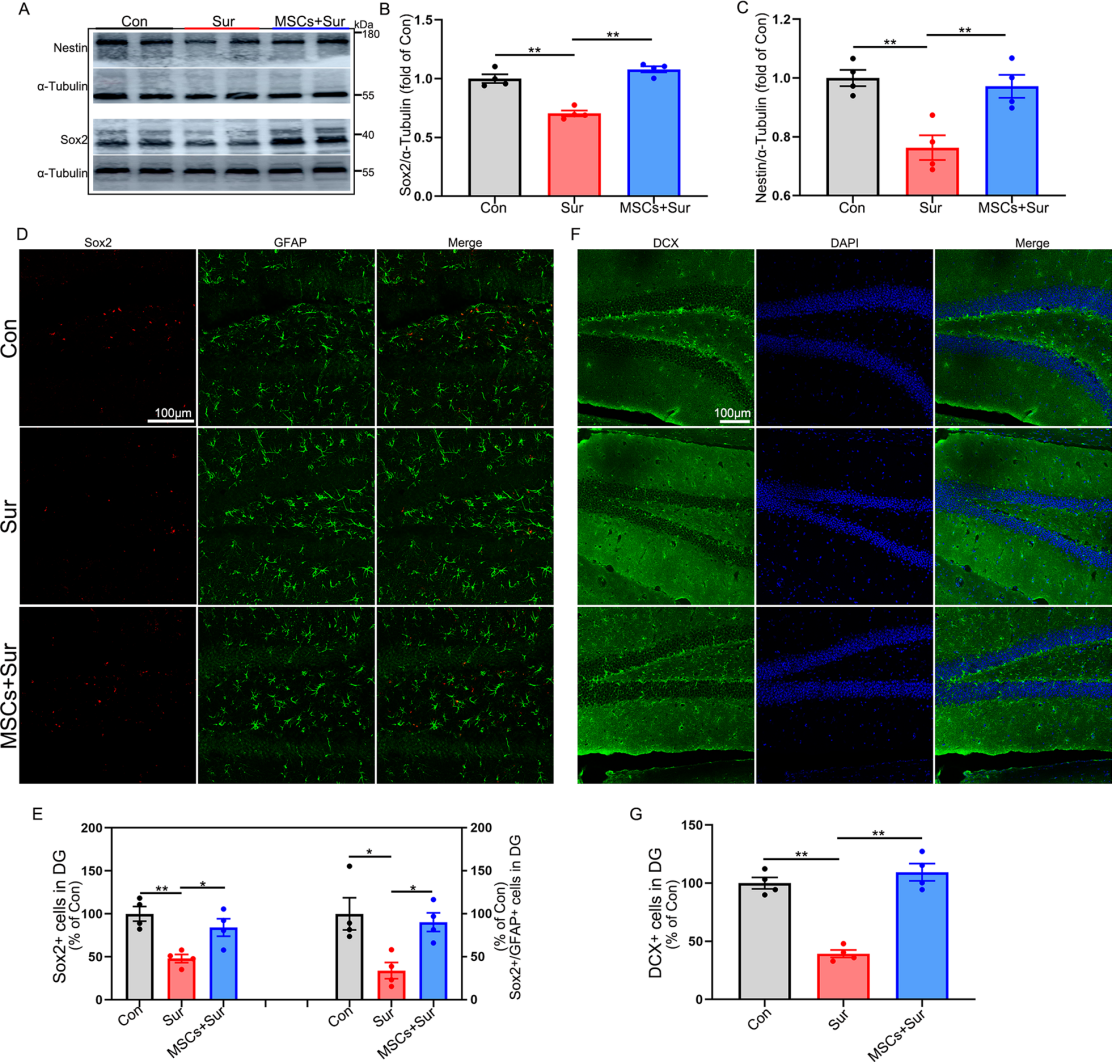
hUC-MSCs reversed the decline of neural stem/progenitor cells and immature neurons in aged mice with PND. Hippocampus/brain was harvested 15 days after surgery for western blotting or immunostaining. **A** Representative Nestin and Sox2 western blotting images of the hippocampus. α-Tubulin was included as a loading control. All full-length blots are presented in Additional file 2: Fig. 4A. **B**, **C** Quantification of western blotting for Nestin and Sox2 expression in the hippocampus. **D** Representative GFAP and Sox2 immunostaining images of the dentate gyrus (DG) region in the hippocampus. **E** Quantification of GFAP and Sox2 positively stained cells in the DG region. (**F** and **G**) Representative DCX immunostaining images and its quantification of DG region in the hippocampus. All data are shown as mean ± S.E.M. (*n* = 4 mice). Con, control; Sur, anesthesia and surgery; hUC-MSCs, human umbilical cord-derived mesenchymal stem cells; DCX, doublecortin; DG, dentate gyrus. *P < 0.05, **P < 0.01

**Fig. 5:**
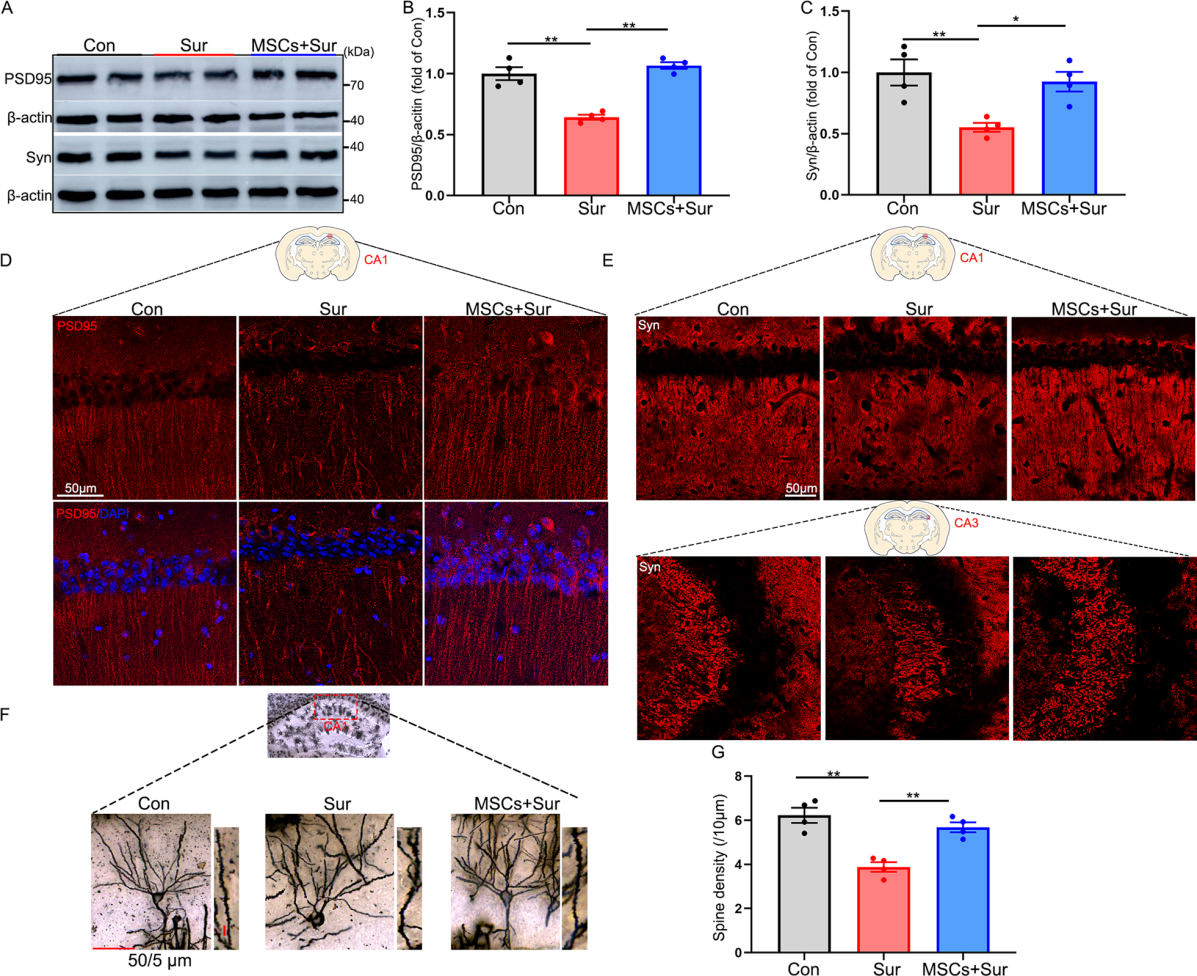
hUC-MSCs restored neuroplasticity dysfunction in aged mice with PND. Hippocampus/brain was harvested 15 days after surgery for western blotting, immunostaining, or Golgi–Cox staining. **A**–**C** Representative PSD95 and Syn western blotting images and their quantifications of the hippocampus. β-actin was included as a loading control. All full-length blots are presented in Additional file 2: Fig. 5A. **D** and **E** Representative PSD95 and Syn immunostaining images in the Cornu Ammonis 1 (CA1) and/or CA3 regions of the hippocampus. **F** Representative Golgi-Cox staining images and **G** quantification of dendritic spine density on tertiary branches in the CA1 region. All data are shown as mean ± S.E.M. (*n* = 4 mice). Con, control; Sur, anesthesia and surgery; hUC-MSCs, human umbilical cord-derived mesenchymal stem cells, PSD95, postsynaptic density protein 95; Syn, synaptophysin; CA1, Cornu Ammonis 1; DG, dentate gyrus. *P < 0.05, **P < 0.01

Endogenous neurogenesis and neuroplasticity are closely associated with learning and memory [12, 28–30]. Impaired hippocampal adult neurogenesis and neuroplasticity underlie the development of PND [16]. However, it is unclear whether hUC-MSC transplantation can reverse and restore this impairment. To determine the effects of hUC-MSCs on endogenous neurogenesis, neural stem/progenitor cells and immature neurons in the hippocampus were analyzed 15 days after surgery according to previous studies [12, 30]. Western blot analysis showed that, in contrast to control mice, anesthesia and surgery inhibited Sox2 [*F*_(2, 9)_ = 44.235, *P* < 0.0001] and Nestin [*F*_(2, 9)_ = 12.389, *P* = 0.003] expression, two markers of neural stem cells, in the hippocampus. hUC-MSC treatment promoted the upregulation of Sox2 and Nestin expression (Fig. 4A–C). Immunostaining results showed a significant decrease in the number of Sox2^+^ [*F*_(2, 9)_ = 10.683, *P* = 0.004], Sox2^+^/GFAP^+^ [*F*_(2, 9)_ = 6.858, *P* = 0.016] and DCX^+^ (a marker of immature neurons) [*F*_(2, 9)_ = 47.764, *P* < 0.0001] in the dentate gyrus (DG) region of the hippocampus in mice with PND, compared with those in control and MSC-treated mice, suggesting that hUC-MSCs reversed the reduction in neurogenesis (Fig. 4D–G). Next, we assessed the effects of hUC-MSC treatment on hippocampal neuroplasticity. Western blot analysis showed that anesthesia and surgery decreased the levels of two key synaptic proteins in the hippocampus, PSD95 [*F*_(2, 9)_ = 38.307, *P* < 0.0001] and synaptophysin (Syn) [*F*_(2, 9)_ = 8.992, *P* = 0.007], which were effectively reversed by hUC-MSC treatment (Fig. 5A–C). Immunostaining for PSD95 and Syn in CA1 and CA3 further confirmed these results (Fig. 5D, E). Neuroplasticity dysfunction in the hippocampus, as reflected by the dendritic spine density, was further confirmed by Golgi–Cox staining. Anesthesia and surgery promoted the loss of dendritic spines, whereas hUC-MSCs significantly attenuated this decrease [*F*_(2, 9)_ = 20.597, *P* < 0.0001] (Fig. 5F, G). Taken together, these data suggest that anesthesia and surgery can impair endogenous neurogenesis and neuroplasticity in the hippocampus. hUC-MSC treatment reverses the detrimental effects of anesthesia and surgery, which may promote the stability of the neural network in the hippocampus.

### hUC-MSCs upregulated hippocampal mature BDNF level but did not affect proBDNF expression level in PND

**Fig. 6:**
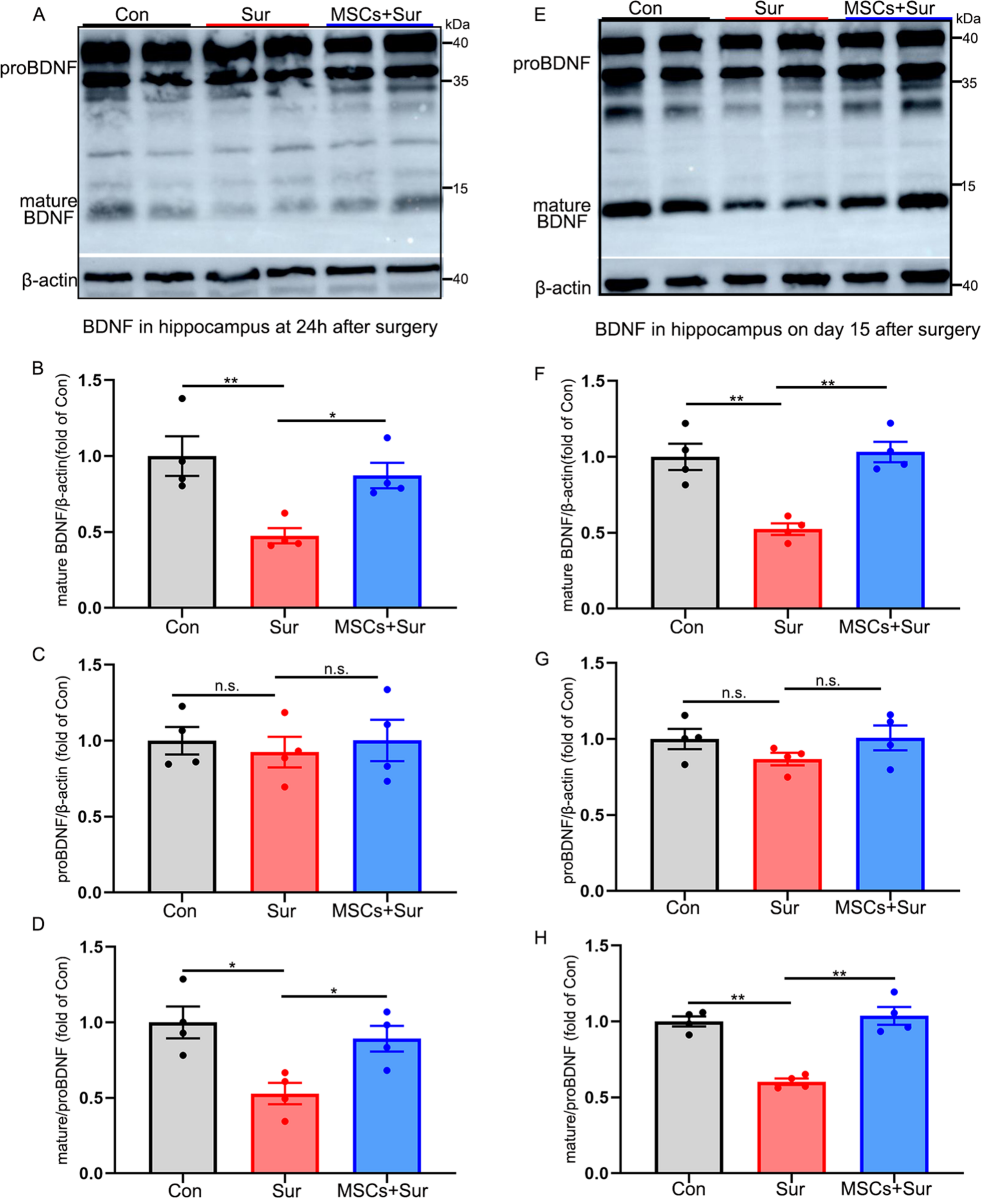
hUC-MSCs inhibited the reduction of hippocampal mature BDNF level but did not affect proBDNF expression level in PND. **A**–**D** Representative mature BDNF and proBDNF western blotting images and quantitative analysis of the hippocampus harvested 24 h after surgery. β-actin was included as a loading control. All full-length blots are presented in Additional file 2: Fig. 6A. **E**–**H** Representative mature BDNF and proBDNF western blotting images and quantitative analysis of the hippocampus harvested 15 days after surgery. β-actin was included as a loading control. All full-length blots are presented in Additional file 2: Fig. 6E. All data are shown as mean ± S.E.M. (*n* = 4 mice). Con, control; Sur, anesthesia and surgery; hUC-MSCs, human umbilical cord-derived mesenchymal stem cells, BDNF, brain-derived neurotrophic factor; n.s., no significant difference. *P < 0.05, **P < 0.01

BDNF abundance is necessary to maintain neurogenesis and neuroplasticity in the hippocampus [31]. Elevated levels of inflammatory cytokines have been reported to induce a significant decrease in BDNF expression [32, 33]. Furthermore, our research and previous studies have shown that the reduction of mature BDNF in the hippocampus may be involved in PND [18, 34]. Therefore, we next investigated the effects of hUC-MSC treatment on BDNF expression, including its precursors (proBDNF, 28–45 kDa) and mature isoform (14 kDa), in the hippocampus at 24 h and 15 days after surgery using western blotting. Consistent with previous findings, anesthesia and surgery significantly decreased the expression level of mature BDNF 24 h [*F*_(2, 9)_ = 8.428, *P* = 0.009] and 15 days [*F*_(2, 9)_ = 17.776, *P* = 0.001] after anesthesia and surgery. However, treatment with hUC-MSCs alleviated this effect (Fig. 6A, B, E, F). Notably, anesthesia and surgery, and hUC-MSC treatment did not significantly affect the level of proBDNF in the hippocampus 24 h or 15 days after surgery and hUC-MSC treatment (*P* > 0.05, Fig. 6A, C, E, G). The ratio of mature BDNF to proBDNF in the hippocampus was also quantified, and a similar trend to that of mature BDNF was observed. Anesthesia and surgery decreased the ratio of mature BDNF to proBDNF either 24 h [*F*_(2, 9)_ = 7.787, *P* = 0.011] or 15 days [*F*_(2, 9)_ = 34.424, *P* < 0.0001], and hUC-MSCs blocked the alterations (Fig. 6D, H). These results indicate that anesthesia and surgery may inhibit BDNF signaling in the hippocampus and that hUC-MSC treatment may improve PND by positively affecting BDNF signaling in aged mice.

### BDNF/TrkB signaling was involved in hUC-MSC-mediated cognitive improvement in aged mice with PND

**Fig. 7:**
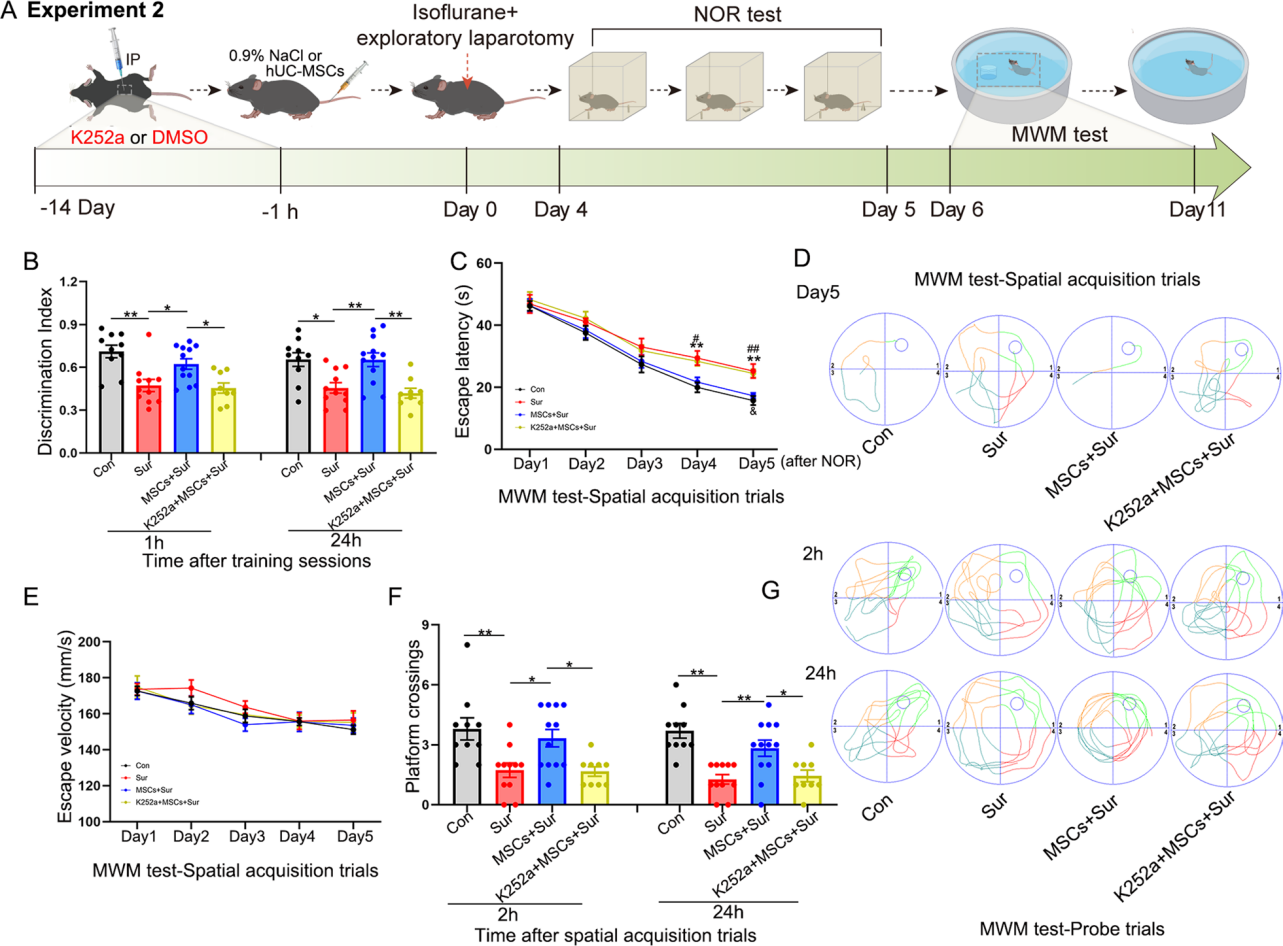
The role of BDNF/TrkB signaling in mediating hUC-MSC-driven amelioration of learning and memory impairment in aged mice with PND. **A** Schematic illustrating the timeline used for K252a (an antagonist of the TrkB receptor) or DMSO, hUC-MSC treatment, isoflurane anesthesia and exploratory laparotomy, NOR and MWM tests in the second experiment. **B** The NOR test was performed to measure recognition memory. **C**–**G** Hippocampus-dependent spatial learning and memory were evaluated by the MWM test consisting of spatial acquisition trials **C**, **D** and **E** and probe trials (**F** and **G**). **C** Escape latency to reach the platform. *P < 0.05 indicates “versus the control groups”, #*P* < 0.05 indicates “versus the MSC plus surgery group”, ^&^*P* < 0.05 indicates “versus the MSCs plus surgery group”. **D** The typical escape routes on day 5 and **E** average swimming speed during the acquisition trials. **F** Average number of platform crossings within a 60-s limit and **G** the typical escape routes in probe trials. All data are shown as mean ± S.E.M. (*n* = 9–12 mice). Con, control; Sur, anesthesia and surgery; hUC-MSCs, human umbilical cord-derived mesenchymal stem cells; MWM, Morris water maze; NOR, novel object recognition; BDNF, brain-derived neurotrophic factor; TrkB, Tropomyosin receptor kinase B; DMSO, dimethyl sulfoxide. *P < 0.05, **P < 0.01

BDNF binds with high affinity to its TrkB receptor and triggers downstream pathways to modulate synaptic and neurogenetic processes [31]. To further determine whether BDNF/TrkB signaling mediates the neuroprotective effects of hUC-MSC treatment on PND in aged mice, mice were intraperitoneally administered K252a for 15 days before hUC-MSC treatment in the following experiment and the same amount of DMSO and normal saline were administered intraperitoneally to mice in other groups. hUC-MSCs were transplanted 1 h before anesthesia, and exploratory laparotomy was performed under isoflurane anesthesia. The NOR and MWM tests were performed 4–11 days after surgery (Fig. 7A). In the NOR test, MSC-treated mice spent more time exploring new objects than mice under anesthesia and surgery, either 1 h [*F*_(3, 38)_ = 8.901, *P* < 0.0001] or 24 h [*F*_(3, 38)_ = 8.328, *P* < 0.0001] after the initial training sessions. However, the intraperitoneal injection of K252a abolished the protective effects of hUC-MSCs (Fig. 7B). Similarly, in the MWM test, the escape latency of MSC-treated mice was significantly lower than that of mice under anesthesia and surgery on day 5 [*F*_(3, 38)_ = 9.483, *P* < 0.0001], and this effect was reversed by intraperitoneal injections of K252a (Fig. 7C). However, K252a did not significantly block the protective effects of hUC-MSCs on PND on day 4 after surgery (*P* = 0.076), although mice in the MSC-treated group showed a significantly shorter escape latency than mice in the anesthesia and surgery group (*P* = 0.016). Typical escape routes on day 5 of the spatial acquisition trials are shown in Fig. 7D. The escape velocity was not significantly different between the groups, excluding potential locomotor impairments (Fig. 7E). In the probe trials, MSC-treated mice exhibited a significant increase in platform crossings within 60 s than that in mice under anesthesia and surgery either 2 [*F*_(3, 38)_ = 6.632, *P* = 0.001] or 24 h [*F*_(3, 38)_ = 11.293, *P* < 0.0001] after the spatial acquisition trials, while K252a decreased the number of mice treated with hUC-MSCs crossing the platform location (Fig. 7F). The typical routes of platform crossings during the probe trials are shown in Fig. 7G. These results suggest that activation of BDNF/TrkB signaling may be implicated in hUC-MSC-mediated cognitive improvement in PND mice.

### Inhibition of BDNF/TrkB signaling weakened the potential of hUC-MSCs to restore impairments of neurogenesis and neuroplasticity in PND mice

**Fig. 8:**
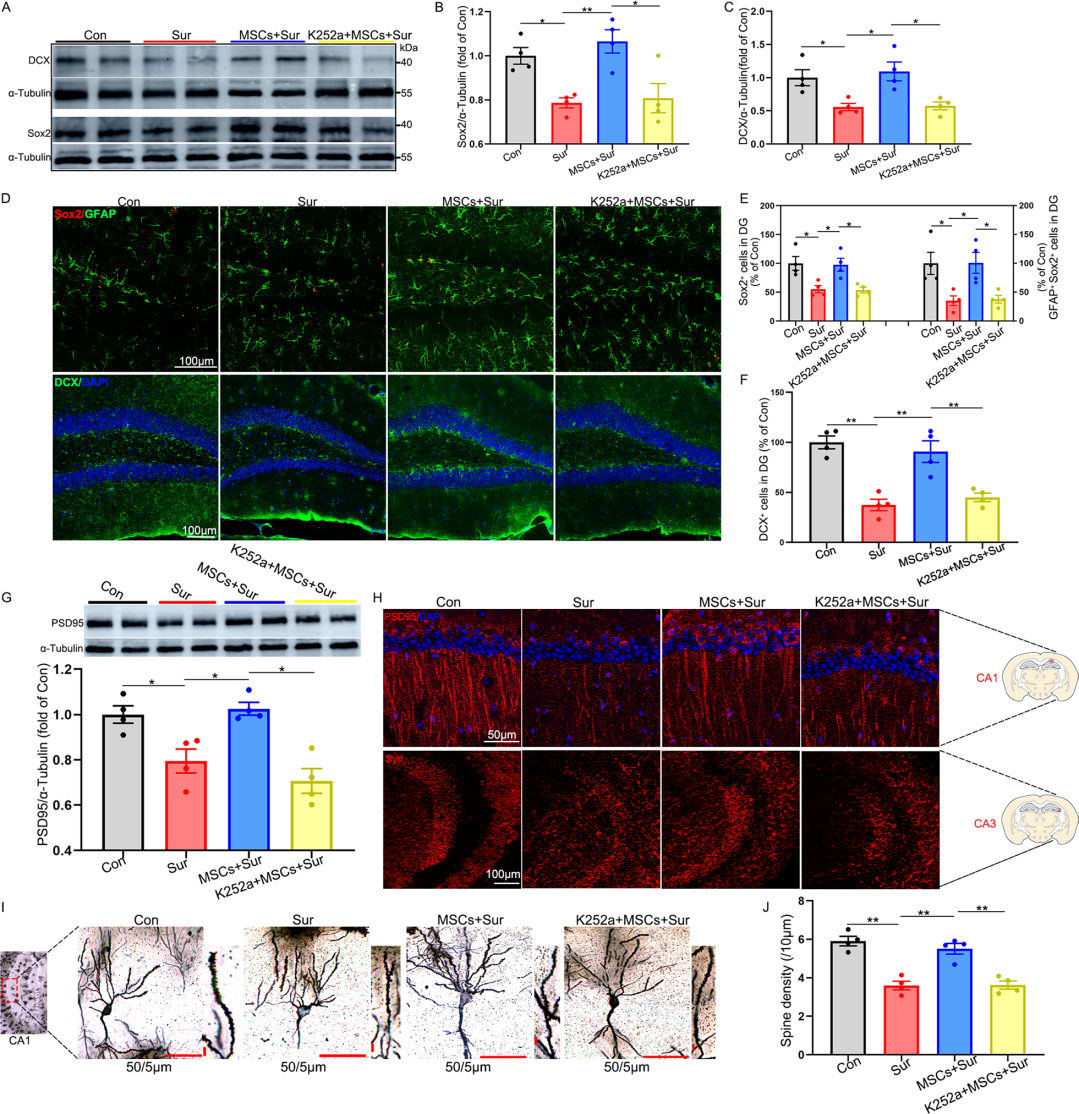
Inhibition of BDNF/TrkB signaling weakened the capacity of hUC-MSCs to restore impairments of neurogenesis and neuroplasticity. Hippocampus/brain was harvested 15 days after surgery for western blotting, immunostaining, or Golgi–Cox staining. **A** Representative Sox2 and DCX western blotting images of the hippocampus. α-Tubulin was included as a loading control. All full-length blots are presented in Additional file 2: Fig. 8A. **B** and **C** Quantification (via western blotting) of Sox2 and DCX expression levels in the hippocampus. **D** Representative GFAP and Sox2, and DCX immunostaining images of the DG region in the hippocampus. **E** Quantification of GFAP and Sox2 positively stained cells in the DG region. **F** Quantification of DCX positively stained cells in the DG region. **G** Representative PSD95 western blotting images and its quantification of the hippocampus. α-Tubulin was included as a loading control. All full-length blots are presented in Additional file 2: Fig. 8G. **H** Representative PSD95 and Syn immunostaining images in the CA1 or CA3 regions of the hippocampus. **I** Representative Golgi–Cox staining images and **J** quantification of dendritic spine density on tertiary branches in the CA1 region. All data are shown as mean ± S.E.M. (*n* = 4 mice). Con, control; Sur, anesthesia and surgery; hUC-MSCs, human umbilical cord-derived mesenchymal stem cells; BDNF, brain-derived neurotrophic factor; TrkB, Tropomyosin receptor kinase B; PSD95, postsynaptic density protein 95; Syn, synaptophysin; DCX, doublecortin; CA1, Cornu Ammonis 1; DG, dentate gyrus *P < 0.05, **P < 0.01

To investigate the effects of BDNF/TrkB signaling on hUC-MSC-mediated endogenous neurogenesis and neuroplasticity in the hippocampus, the hippocampus/brain was harvested 15 days after surgery. Western blotting results from the endogenous neurogenesis analysis showed that, in contrast to that observed in mice under anesthesia and surgery, hUC-MSC treatment promoted Sox2 [*F*_(3, 12)_ = 8.448, *P* = 0.003] and DCX [*F*_(3, 12)_ = 7.558, *P* = 0.004] expression in the hippocampus. However, intraperitoneal injections of K252a abolished the therapeutic effects of hUC-MSCs (Fig. 8A–C). Immunostaining results showed a significant increase in the number of Sox2^+^ [*F*_(3, 12)_ = 7.846, *P* = 0.004], Sox2^+^/GFAP^+^ [*F*_(3, 12)_ = 6.689, *P* = 0.007] and DCX [*F*_(3, 12)_ = 19.063, *P* < 0.0001] in the DG region of the hippocampus in MSC-treated mice compared with that in mice with PND. However, K252a treatment reversed the protective effects of hUC-MSCs on neural progenitor cells and immature neurons (Fig. 8D–F). Next, we assessed the effect of BDNF/TrkB signaling on the hUC-MSC-mediated neuroplasticity improvement in the hippocampus. Western blot analysis showed that, in contrast to that observed in mice under anesthesia and surgery, hUC-MSC treatment promoted the expression of PSD95 in the hippocampus [*F*_(3, 12)_ = 12.052, *P* = 0.001], which were blocked by intraperitoneal injections of K252a (Fig. 8G). Immunostaining for PSD95 and Syn visually showed a decrease in the two key synaptic proteins in the CA1 and CA3 (Fig. 8H). Furthermore, hUC-MSC treatment significantly increased the density of dendritic spines as assessed by Golgi–Cox staining, and K252a blocked these alterations [*F*_(3, 12)_ = 25.668, *P* < 0.0001] (Fig. 8I, J). Together, these results suggest that the activation of BDNF/TrkB signaling may be a mechanism for hUC-MSC-induced neuroprotection against impairments in neurogenesis and neuroplasticity in PND.

### The BDNF-TrkB-CREB axis may be involved in the therapeutic effects of hUC-MSCs on PND in aged mice

**Fig. 9:**
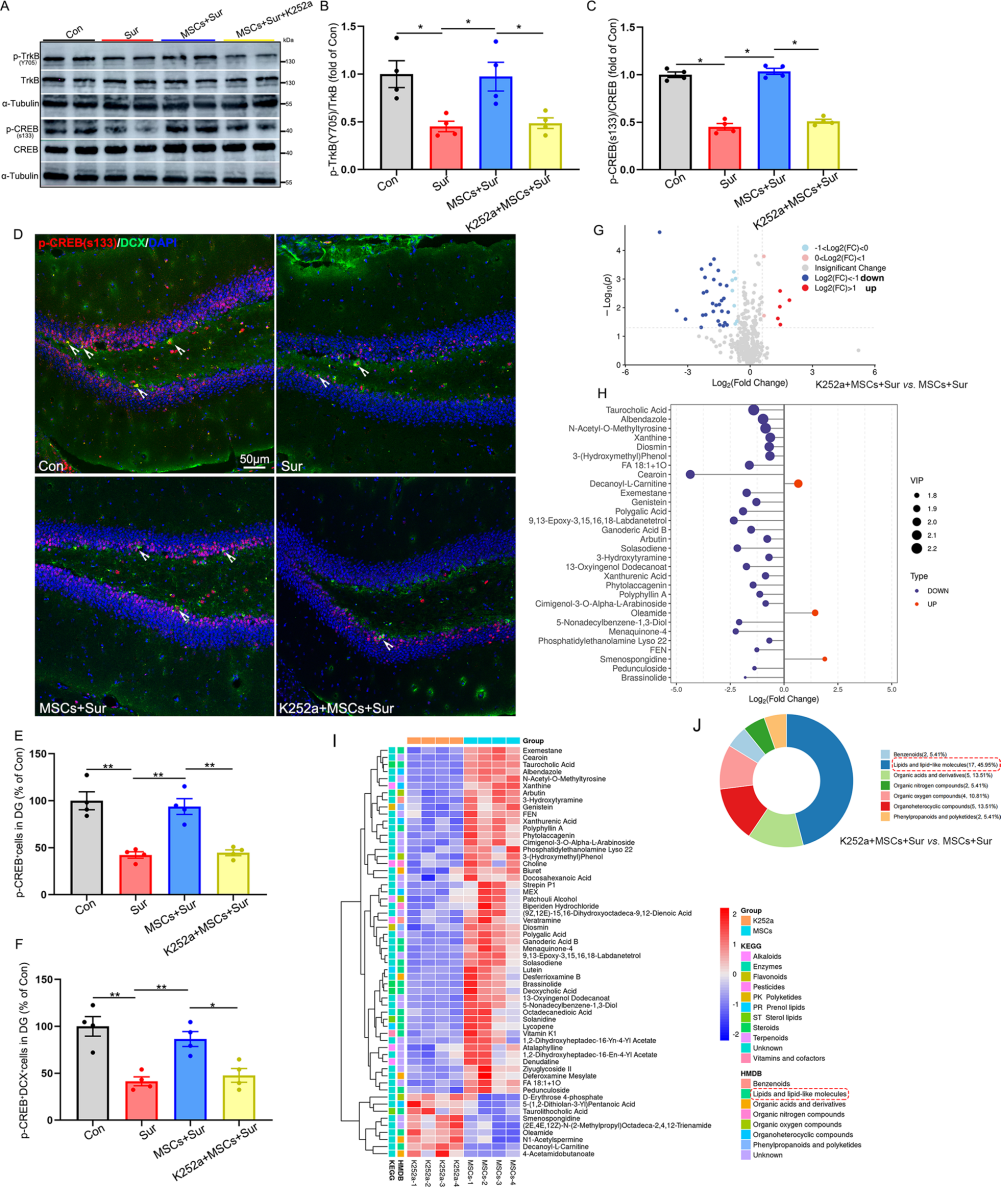
BDNF/TrkB signaling increased the phosphorylation of CREB and regulated neurogenesis and lipid metabolism in hUC-MSC-treated PND mice. Hippocampus/brain was harvested 15 days after surgery for western blotting or immunostaining. **A** Representative TrkB, phosphor (p)-TrkB, CREB, and p-CREB western blotting images of the hippocampus. α-Tubulin was included as a loading control. All full-length blots are presented in Additional file 2: Fig. 9A. **B** and **C** Quantitative analysis of p-TrkB/TrkB and p-CREB/CREB in the hippocampus. **D** Representative DCX and p-CREB immunostaining images of the DG region in hippocampus. **E** Quantification of p-CREB positively stained cells in the DG region. **F** Quantification of DCX and p-CREB positively stained cells in the DG region. **G** Volcano plot between MSCs plus surgery group (MSCs + Sur) and K252a plus MSCs plus surgery group (K252a + MSCs + Sur) from the 8 hippocampal tissue samples. **H** Differentially expressed metabolites were identified. **I** Heatmap showing hierarchical clustering of metabolites and their classification in KEGG and HMDB databases. **J** The classification of differentially expressed metabolites in the HMDB database. All data are shown as mean ± S.E.M. (*n* = 4 mice). Con, control; Sur, anesthesia and surgery; hUC-MSCs, human umbilical cord-derived mesenchymal stem cells; BDNF, brain-derived neurotrophic factor; TrkB, Tropomyosin receptor kinase B; CREB, cAMP response element-binding protein; DCX, doublecortin; DG, dentate gyrus *P < 0.05, **P < 0.01

We further explored the potential mechanism by which BDNF/TrkB signaling mediates hUC-MSCs attenuating cognitive improvement in PND mice. Given that the activation of CREB initiates the transcription of genes associated with neurogenesis and that BDNF/TrkB signaling is a potential upstream regulator of CREB [30, 35], we speculated that the activation of the BDNF-TrkB-CREB axis may underlie the protective effects of hUC-MSCs on cognitive function in aged mice with PND. We directly examined the CREB phosphorylation 15 days after surgery following hUC-MSC treatment and K252a injections. The results showed that anesthesia and surgery significantly inhibited the phosphorylation of both the TrkB receptor and CREB in the hippocampus, and hUC-MSC treatment promoted the upregulation of the phospho (p)-TrkB receptor [*F*_(3, 12)_ = 7.432, *P* = 0.004] and p-CREB [*F*_(3, 12)_ = 8.801, *P* = 0.002]. K252a abolished the hUC-MSC-induced increases in p-TrkB and p-CREB, indicating that hUC-MSC treatment evoked the activation of the BDNF/TrkB/CREB signaling pathway (Fig. 9A–C). To further confirm that the BDNF/TrkB/CREB signaling pathway is involved in the therapeutic effects of hUC-MSCs on PND, we examined the p-CREB levels in immature neurons using DCX^+^ immunostaining at 15 days after surgery. As shown in Fig. 9D–F, weak p-CREB signals were observed in the DG region of the hippocampus in the anesthesia and surgery groups, and the number of DCX^+^ and p-CREB^+^ cells was dramatically reduced after anesthesia and surgery compared with that in the control group. However, hUC-MSC treatment attenuated the downregulation of p-CREB [*F*_(3, 12)_ = 20.975, *P* < 0.0001] and increased the number of DCX^+^ and p-CREB^+^ cells [*F*_(3, 12)_ = 13.361, *P* < 0.0001] in the DG, suggesting that the BDNF/TrkB/CREB signaling pathway may mediate the neurogenesis and neuroprotective effects of hUC-MSCs in PND mice. Furthermore, blocking TrkB receptor activation with K252a altered the profiles of metabolites in the hippocampus at 15 days after surgery (Fig. 9G). The metabolites with most changes by K252a injections are displayed in Fig. 9H. Among them, lipids and lipid-like molecules were significantly involved, indicating that the BDNF/TrkB/CREB signaling pathway is likely to regulate lipid metabolism and exert a neuroprotective effect on PND (Fig. 9I, J).

## Discussion

**Fig. 10:**
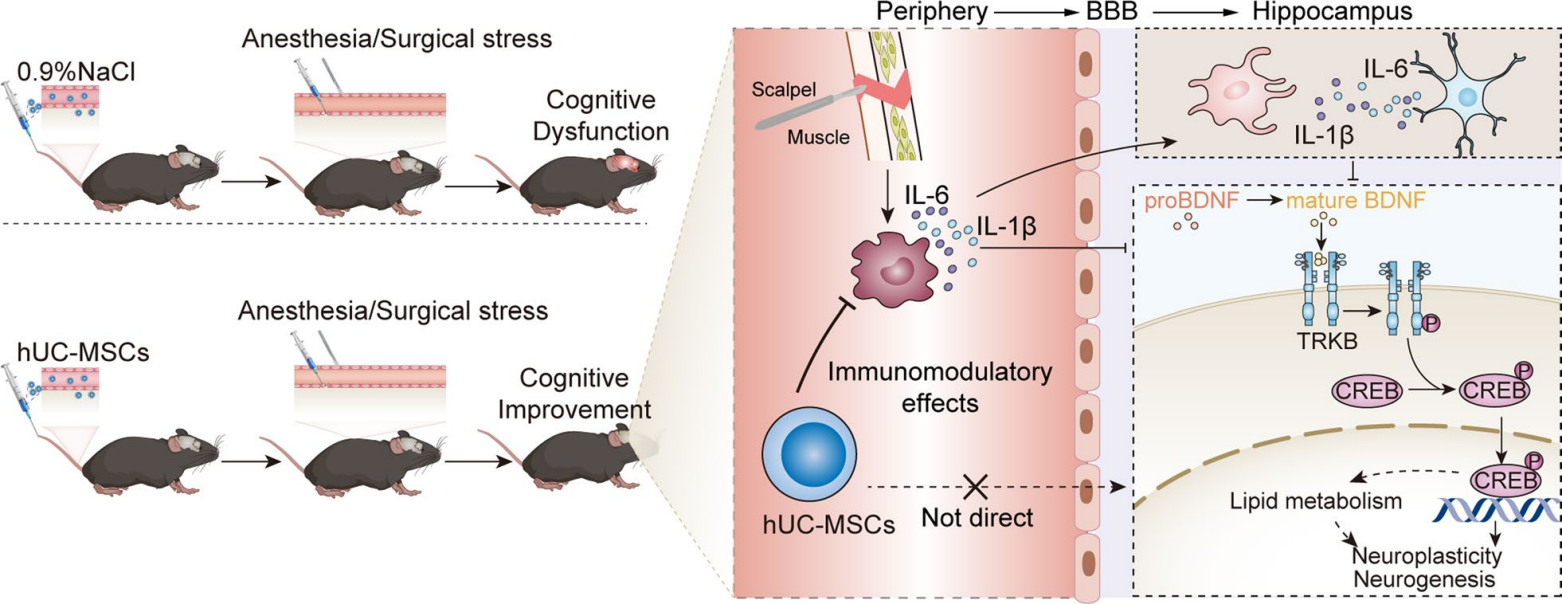
Working hypothesis for hUC-MSC-mediated therapeutic effect on PND in aged mice. Anesthesia and surgery initiate peripheral inflammatory responses and promote release of systemic pro-inflammatory cytokines; these cytokines can infiltrate the blood–brain barrier (BBB) and activate the microglia and astrocytes to produce a wide range of inflammatory cytokines and negatively affect the BDNF/TrkB/CREB signaling pathway, leading to the impairment of the endogenous neurogenesis and neuroplasticity. hUC-MSC treatment inhibits early systemic inflammatory responses to anesthesia and surgery and further down-regulates neuroinflammatory responses and restores neurogenesis and neuroplasticity dysfunction by activating the BDNF/TrkB/CREB signaling pathway and modulation of its potential downstream effect on lipid metabolism. The combined mechanisms contribute to cognitive improvement in aged mice with PND. hUC-MSCs, human umbilical cord-derived mesenchymal stem cells; BDNF, brain-derived neurotrophic factor; TrkB, Tropomyosin receptor kinase B; CREB, cAMP response element-binding protein. BBB, blood–brain barrier

To the best of our knowledge, this is the first study to explore hUC-MSCs for PND in aged mice. Our results revealed that hUC-MSC treatment had beneficial effects on learning and memory impairment following anesthesia and surgery, as evidenced by better cognitive performance in the MWM and NOR tests. Moreover, we demonstrated that hUC-MSCs could inhibit early systemic inflammatory responses to surgery and attenuate PND-related hippocampal inflammation and restore impaired endogenous neurogenesis and neuroplasticity. We further identified that the BDNF/TrkB/CREB signaling pathway may, at least in part, be a potential mechanism underlying the neuroprotective effect of hUC-MSCs by regulating lipid metabolism (Fig. 10).

Cognitive impairment during the perioperative period was first recognized 100 years ago, and in older patients, postoperative cognitive recovery remains a concern [7]. The consensus document on the assessment of cognitive change associated with anesthesia and surgery was published in 2018 by the International Nomenclature Consensus Working Group where the term “perioperative neurocognitive disorder” is widely applied to describe any objective cognitive change in the perioperative period [2]. An improved PND animal model is a key feature of this study. Previously reported surgical procedures conducted in rodents include laparotomy, vascular exposure, splenectomy, cecectomy and appendectomy, partial hepatectomy, and tibial fracture [36]. Organectomy may not be the best choice, as this procedure affects homeostasis and affects general health, exerting a significant impact on PND. For example, the spleen modulates the innate immune response, and the liver is important for metabolic regulation. Exploratory laparotomy was selected as the surgical procedure in this study because it is routinely performed in older patients and does not impair limb functions, such as tibial fracture, thus ensuring normal activities in behavioral tests. Our results showed that exploratory laparotomy under isoflurane anesthesia significantly impaired learning and memory in aged mice, as evidenced by a longer latency, fewer platform crossings in the MWM test, and a lower discrimination index in the NOR test. Notably, swimming speed did not differ between the groups, suggesting that the activities of the mice returned to normal 6 days after surgery.

Over the past few decades, despite substantial investments through small molecules or targeting inflammation-based therapies in preclinical studies, pharmacological treatments have had little success, partly because there is a lack of understanding of the key pathogenic mechanisms underlying PND; to date, no single molecular pathway to modulate disease progression has been identified [3, 7, 8]. MSC treatment may provide a biological treatment alternative to traditional pharmacological therapies. MSCs derived from human umbilical cord, which is considered biological waste, are preferred because of their greater differentiation ability and secretory reactions. hUC-MSCs can exert an effect on immunomodulation against systemic inflammatory responses and brain pathology or secrete a wide range of functional factors in vivo, including growth factors, cytokines, chemokines, and metabolites to induce endogenous stem cells activity and differentiate into the required cell types, which can replace damaged tissue or restore tissue homeostasis [10, 11]. To date, cognitive improvement has been reported in various models of neurodegenerative diseases following hUC-MSC transplantation in rodent models [10, 12, 37]. Importantly, several clinical trials have also demonstrated the safety and efficacy of hUC-MSCs for the treatment of stroke and Parkinson’s disease patients with amelioration of disease symptoms and slower disease progression observed following hUC-MSC injection [38]. Our results revealed that mice that underwent hUC-MSC transplantation exhibited a better performance in the MWM and NOR tests than mice with PND. These results provide direct evidence that perioperative administration of hUC-MSCs may be a promising therapy for the prevention and treatment of PND.

Chronic low-grade neuroinflammation increases the vulnerability of the aging brain to external insults [39]. Exposure to surgery and anesthesia can activate peripheral immune cells and trigger systemic inflammation, which then infiltrate the brain across the damaged blood–brain barrier and activate resident microglia to drive cognitive deficits [3, 4, 13]. Microglia are the key cell type affected by surgical trauma and anesthesia and are central players in regulating neuroinflammation. On activation, microglia exhibit distinct morphologic changes, release proinflammatory factors and contribute to the subsequent activation of astrocytes [3, 40]. The role of IL-1β and IL-6 in PND is also well characterized. A peripheral surgery-induced innate immune responses and consequent release of inflammatory cytokines can trigger an IL-1β-mediated neuroinflammatory process that underlies memory impairment [41]. Furthermore, increased IL-6 levels after surgery were found to lead to postoperative cognitive impairment through IL-6 trans-signaling in mouse CA1 neurons [42]. hUC-MSCs administered intravenously are most trapped in the lungs and hard to reach the brain despite increased blood–brain barrier permeability in PND [3, 43]. We did not detect hUC-MSCs in brain parenchyma 7 days and 14 days after surgery by staining human nuclei using immunofluorescence (MAB1281) and measuring human-specific DNA using PCR (Additional file 1: Fig. S1), which is consistent with a recent study that investigated the effects of intravenously administered hUC-MSCs on schizophrenia [43]. However, systemically administered MSCs have been increasingly focused on its immunomodulatory roles. hUC-MSCs can exert an immunomodulatory effect in the periphery to manipulate glia-mediated neuroinflammation [14]. Recent studies have shown that hUC-MSCs intravenously or intraperitoneally transplanted can suppress microglia activation and neuroinflammatory responses in a paracrine manner [12, 43]. Here, we comprehensively evaluated the effects of hUC-MSCs on the systemic and neuroinflammatory responses in PND model. We found that hUC-MSC treatment significantly prevented surgical trauma-induced peripheral inflammatory responses and subsequent overactivation of astrocytes and microglia, resulting in elevated expression levels of IL-1β and IL-6 in the hippocampus, indicating a potential immunomodulatory effect of hUC-MSCs on PND.

Structural modifications in the brain are closely associated with neuroplasticity and neurogenesis, which underlie dynamic changes in neuronal circuits and are fundamental to learning and memory [44, 45]. Previous studies have suggested an association between PND and neuroplasticity dysregulation [16, 18]. Consistent with these reports, we observed a significant decrease in PSD95 and synaptophysin levels in the hippocampus. Synaptophysin is an integral membrane protein of small synaptic vesicles, and PSD95 is mainly localized in the postsynaptic density of asymmetric synapses. The two proteins have been identified as useful markers for neuroplasticity [46]. Spine density is a morphological feature that is closely associated with neuroplasticity and has been identified as a useful marker of neuroplasticity. We also observed a significant decrease in spine density in the CA1 region of the hippocampus after anesthesia and surgery. Importantly, MSC treatment has been found to promote neuroplasticity [28]. Our results show that hUC-MSC administration prevented the postoperative decreases in PSD95, synaptophysin, and spine density in the hippocampus. Neurogenesis has also been demonstrated to be associated with maintaining neuroplasticity and restoring cognition. Sox2, which is expressed in proliferating astrocytes in the developing brain, is a well-known marker of neural stem cells and is involved in the maturation and differentiation of developing astrocytes [12, 47]. Nestin is a cytoskeletal protein classified as an intermediate filament and another marker of neural stem cells [48]. Nestin^+^ cells in the brain can form neurospheres ex vivo and generated differentiated cells of neuronal and astrocytic lineages. The expression of DCX, a marker for immature neurons, can reflect the number of immature neurons [30]. Importantly, these neurons are under non-pathological conditions and can mature into “fully-grown” neurons [49]. Recent studies have shown that surgery could significantly reduce the number of neural stem cells and immature neurons, which is associated with PND [50–52]. Therefore, to explore whether hUC-MSC treatment affected endogenous neurogenesis, the expression of Sox2, Nestin, and DCX in the hippocampus and also coronal hippocampal sections of mice for DCX^+^ and Sox2^+^/GFAP^+^ stem cells were analyzed according to previous studies [12, 30]. The present study demonstrates a significant decrease in hippocampal neurogenesis in PND and suggests that hUC-MSC treatment reverses these deficits in aged mice. Together, our findings indicate that MSCs derived from the human umbilical cord may reverse cognitive impairment after anesthesia and surgery by restoring neuroplasticity and neurogenesis dysfunction.

BDNF is a neuropeptide whose abundance is essential for neuroplasticity and neurogenesis [31]. BDNF is widely distributed throughout the brain but is highly expressed in the hippocampus and cortex [53]. BDNF is initially synthesized in the cell bodies of neurons and glia as a precursor proBDNF, which is cleaved into mature BDNF [31]. BDNF protein synthesis can be suppressed by pro-inflammatory cytokines, such as IL-1β [32, 33, 54]. Mature BDNF is released presumably by a Ca2^+^-dependent mechanism and works by binding with high affinity to the TrkB receptor for neuronal survival, differentiation, functions, and neuroplasticity [31, 53]. However, proBDNF plays an opposing role mediating neuronal apoptosis, inhibiting cellular proliferation, and contributing to long-term depression [17]. Although proBDNF evaluation was not included in previous studies, we have shown that mature BDNF may play a critical role in PND because mature upregulation of BDNF by neuroprotective agents can rescue cognitive impairment after surgery and anesthesia. Consistent with the role of pro- and mature BDNF in cognitive function, we showed that mature BDNF, but not proBDNF, was reduced in the hippocampus after surgery and anesthesia and hUC-MSCs treatment promoted the conversion of proBDNF to mature BDNF. The activation of BDNF/TrkB signaling is important for neuronal function and survival and has been shown to be a key player in the pathogenesis of PND [17, 18]. In support of the role of BDNF/TrkB signaling in hUC-MSC-mediated beneficial effects, intraperitoneal injections of k252a, a TrkB receptor inhibitor, worsened learning and memory in aged mice that underwent hUC-MSC treatment. More importantly, k252a partly abolished the hUC-MSC-mediated restoration of neuroplasticity and endogenous neurogenesis. A K252a intraperitoneal injection was selected over an intracerebroventricular injection because K252a is a small-molecular compound that crosses the blood–brain barrier and may easily redistribute to other tissues and organs, thereby decreasing the concentration of k252a in the brain, when administered by a low concentration and single intracerebroventricular injection [25, 27]. Given that activation of TrkB by mature BDNF can trigger several downstream pathways, we explored the role of CREB, a key molecule for the maintenance and activity of synapses, neurogenesis, and cognition [30], in hUC-MSC-mediated neuroprotection on PND. Importantly, CREB has been identified as a downstream target of BDNF/TrkB, and CREB activation has been shown to modulate lipid metabolism, regulate neuronal maturation, support neuronal survival and synaptogenesis, and contribute to the formation of long-term potentiation and memory in several neuropsychiatric disorders [30, 35, 55–58]. In the present study, hUC-MSC treatment induced activation of the BDNF/TrkB signaling and resulting in increased phosphorylation of CREB in immature neurons. However, k252a administration reversed these changes. Furthermore, we identified lipid metabolism as a potential downstream of this signaling pathway in hUC-MSC-mediated neuroprotection on PND using untargeted metabolomic. These findings support the critical role of the BDNF/TrkB/CREB signaling pathway after hUC-MSC treatment on PND.

This study has significant clinical implications. Despite various sources of MSCs, hUC-MSCs can facilitate the translation of basic science to the perioperative applications because human umbilical cord is considered to be biological waste and its use is associated with fewer ethical concerns, and MSCs derived from the umbilical cord are readily accessible and non-invasive. If hUC-MSC transplantation is proven to be effective in humans, older patients undergoing surgery at a high risk of PND may benefit from hUC-MSC treatment. Also, our findings suggest that the BDNF/TrkB/CREB signaling pathway plays an important role in the pathogenesis of PND and hUC-MSC-mediated neuroprotective effects. Therefore, the activation of this pathway may synergistically enhance the therapeutic effects of hUC-MSCs. Our study had several limitations. First, despite their attractive therapeutic potential, the successful clinical application of hUC-MSCs is hampered by high variability in the therapeutic efficacy of hUC-MSCs because of the diversity and heterogeneity of isolated cells [30]. Second, hUC-MSCs have a limited life span and their potency is reduced after replicative senescence. In the current study, we only evaluated a single-dose and short-term therapeutic effects of hUC-MSCs, whereas PND can persist for a long time after surgery. Further studies are required to optimize dosages and therapeutic time window for the translation of hUC-MSCs to patients with PND. Third, although we have demonstrated the potential downstream effects of hUC-MSC treatment on PND, we have not determined which signaling or bioactive components, such as hepatocyte growth factor [12], mediated by hUC-MSCs work as key triggers to induce these effects.

## Conclusions

The findings of this study suggest that hUC-MSCs can alleviate cognitive impairment after anesthesia and surgery. This effect results from the suppression of systemic and neuroinflammatory responses and restoration of neurogenesis and neuroplasticity dysfunction via the BDNF/TrkB/CREB signaling pathway and its potential downstream effect on lipid metabolism. This study provides a foundation the use of hUC-MSCs in the perioperative period as a treatment for PND in humans.

### Supplementary Information

**Additional file1**. **Figure S1:** Quantification of the PCR results for human-specific DNA in mouse brain.

**Additional file2**. **Figure 4A**: Original western blot gels of Nestin and Sox-2 in the hippocampus. α-Tubulin was included as a loading control. **Figure 5A**: Original western blot gels of PSD95 and Syn in the hippocampus. β-actin was included as a loading control. **Figure 6A** and **E**: Original western blot gels of BDNF (proBDNF, mature BDNF) in the hippocampus. β-actin was included as a loading control. **Figure 8A**: Original western blot gels of DCX and Sox-2 in the hippocampus. α-Tubulin was included as a loading control. **Figure 8G**: Original western blot gels of PSD95 in the hippocampus. α-Tubulin was included as a loading control. **Figure 9A**: Original western blot gels of pTrkB/TrkB and pCREB/CREB in the hippocampus. α-Tubulin was included as a loading control.

## Data Availability

The metabolomic data used and analyzed during the current study are publicly available in the MetaboLights repository at https://www.ebi.ac.uk/metabolights/MTBLS7759. Other datasets used and analyzed during the current study are available from the corresponding author upon reasonable request.

## Abbreviations

PND: Perioperative neurocognitive disorder
hUC-MSCs: Human umbilical cord-derived mesenchymal stromal cells
NOR: Novel object recognition
MWM: Morris water maze
Iba-1: Ionized calcium-binding adaptor molecule-1
GFAP: Glial fibrillary acidic protein
DCX: Doublecortin
PSD95: Postsynaptic density protein 95
ELISA: Enzyme-linked immunosorbent assay
IL-1β: Interleukin-1β
CA1: Cornu ammonis-1
BDNF: Brain-derived neurotrophic factor
TrkB: Tropomyosin receptor kinase B
DG: Dentate gyrus
CREB: CAMP response element-binding protein
