# Supplementary material for: Human umbilical cord-derived mesenchymal stem cells ameliorate perioperative neurocognitive disorder by inhibiting inflammatory responses and activating BDNF/TrkB/CREB signaling pathway in aged mice
Source: Stem Cell Res Ther. 2023 Sep 21;14:263. doi: 10.1186/s13287-023-03499-x (PMC10512658; doi:10.1186/s13287-023-03499-x)
Supplement: Supplementary file 1 — Additional file1. Figure S1: Quantification of the PCR results for human-specific DNA in mouse brain. [file 13287_2023_3499_MOESM1_ESM.pdf]

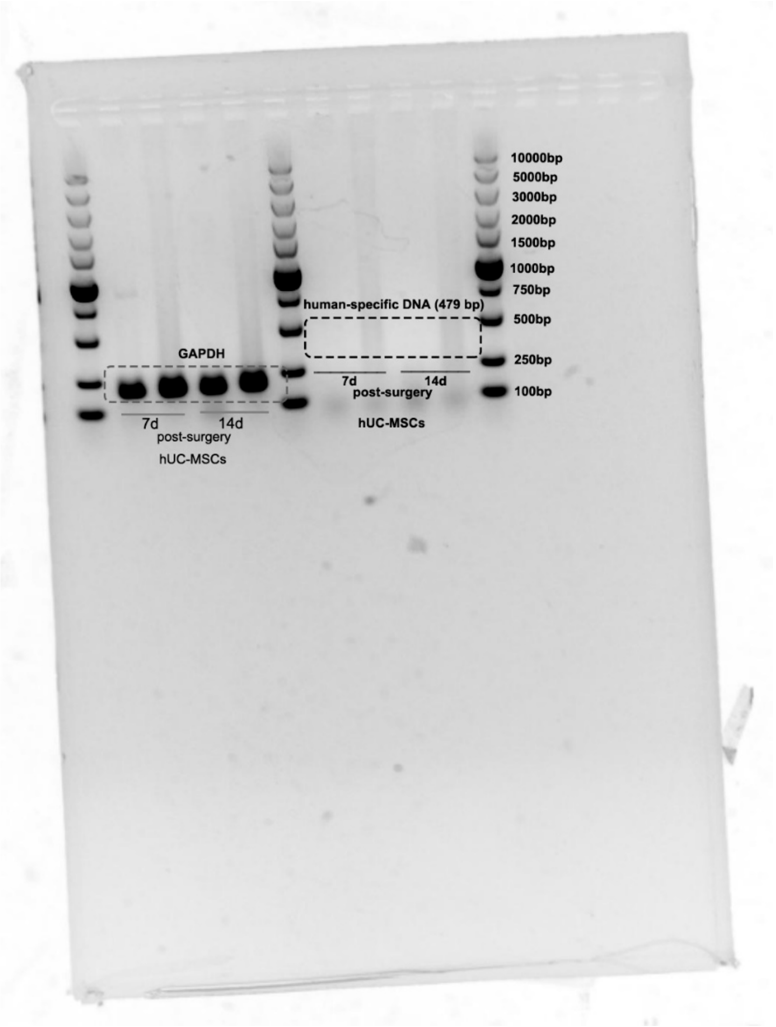

Supplementary Figure S1: Quantification of the PCR results for human-specific DNA in mouse brain. Human-specific DNA in the mouse brain by PCR analysis was used to ensure the homing of intravenously transplanted hUC-MSCs as previously described [1, 2]. We did not detect human-specific DNA in brain derived from mice subjected to hUC-MSCs 7 days and 14 days after surgery. Human-specific DNA (a 479-bp fragment of a highly repetitive α-satellite DNA sequence of the centromere region of human chromosome): forward-5′-GGGATAATTTCAGCTGACTAAACAG-3′, reverse-5′-AAACGTCCACTTGCAGTTCTAG-3′; GAPDH: forward-5′-GGTGAAGGTCGGTGTGAAC-3′, reverse-5′-CTCTGACCTGTGCCGTTGAA-3′

References

1.Guan F, Huang T, Wang X, Xing Q, Gumpper K, Li P, et al.Correction to: The TRIM protein Mitsugumin 53 enhances survival and therapeutic efficacy of stem cells in murine traumatic brain injury. Stem Cell Res Ther.2021;12:522.

2.Cui Y, Ma S, Zhang C, Cao W, Liu M, Li D, et al.Human umbilical cord mesenchymal stem cells transplantation improves cognitive function in Alzheimer's disease mice by decreasing oxidative stress and promoting hippocampal neurogenesis. Behav Brain Res.2017;320:291-301.
