# Supplementary material for: Human umbilical cord-derived mesenchymal stem cells ameliorate perioperative neurocognitive disorder by inhibiting inflammatory responses and activating BDNF/TrkB/CREB signaling pathway in aged mice
Source: Stem Cell Res Ther. 2023 Sep 21;14:263. doi: 10.1186/s13287-023-03499-x (PMC10512658; doi:10.1186/s13287-023-03499-x)

Raw data of Western blot: Full, uncropped gel and blot images

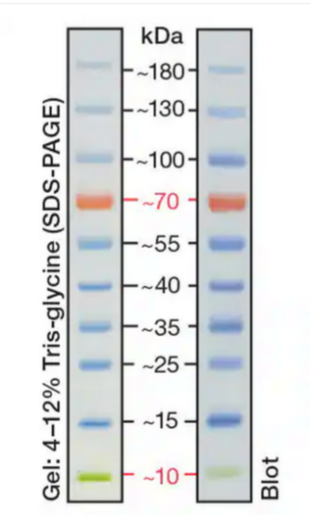

Thermo Scientific PageRuler 26616

Protein Ladder used in the study.

Figure 4A: Original western blot gels of Nestin and Sox-2 in the hippocampus. α-Tubulin was included as a loading control.

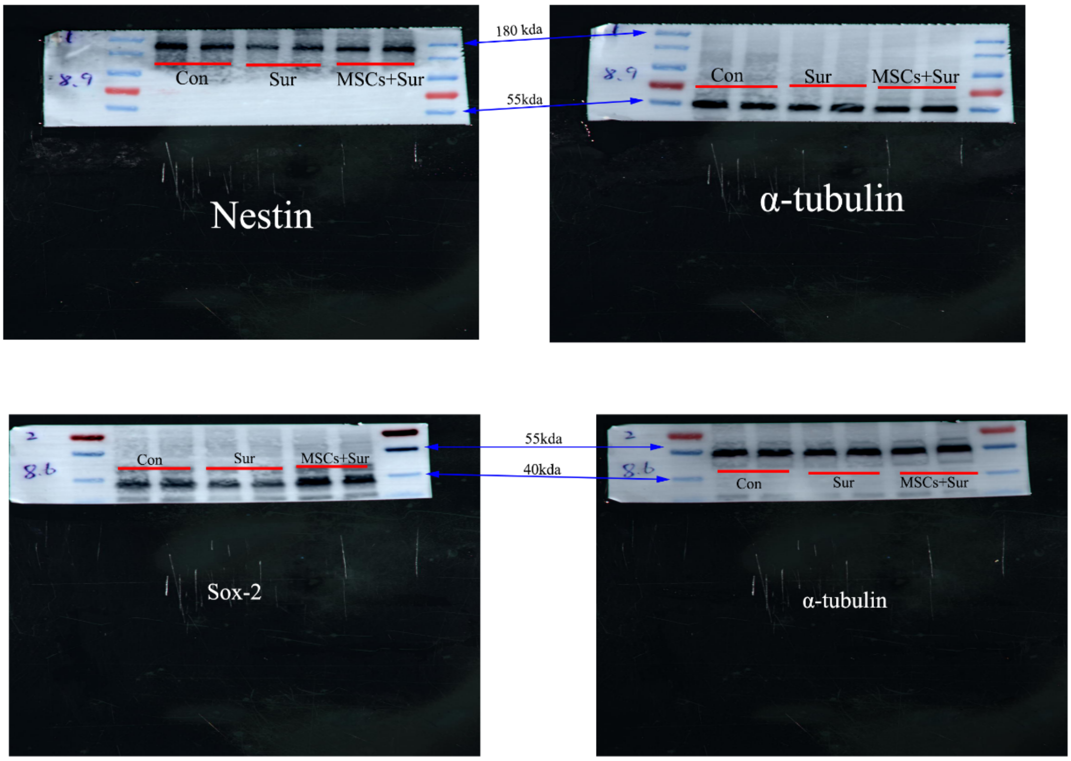

Figure 5A: Original western blot gels of PSD95 and Syn in the hippocampus. β-actin was included as a loading control.

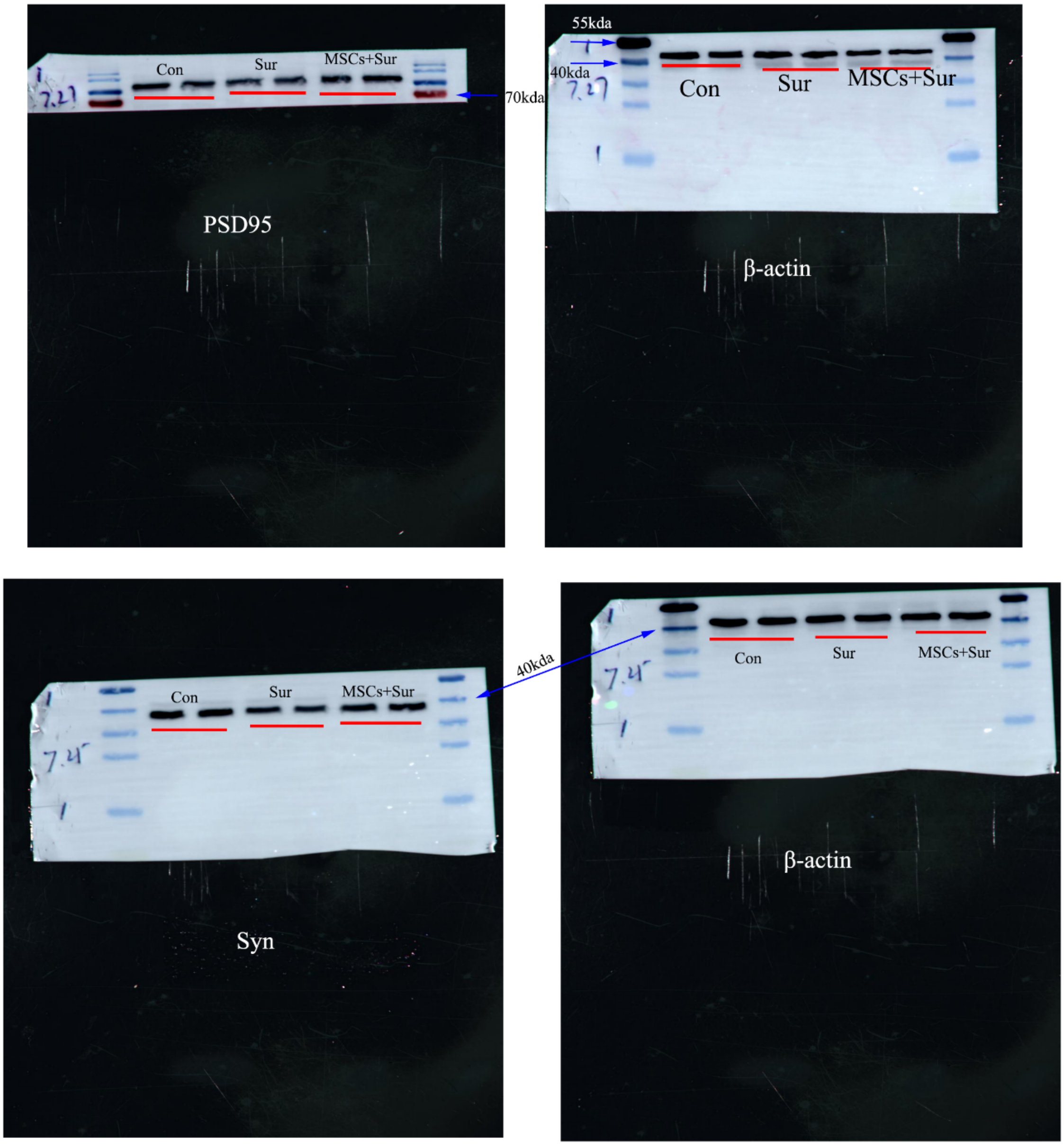

Figure 6 A and E: Original western blot gels of BDNF (proBDNF, mature BDNF) in the hippocampus. β-actin was included as a loading control

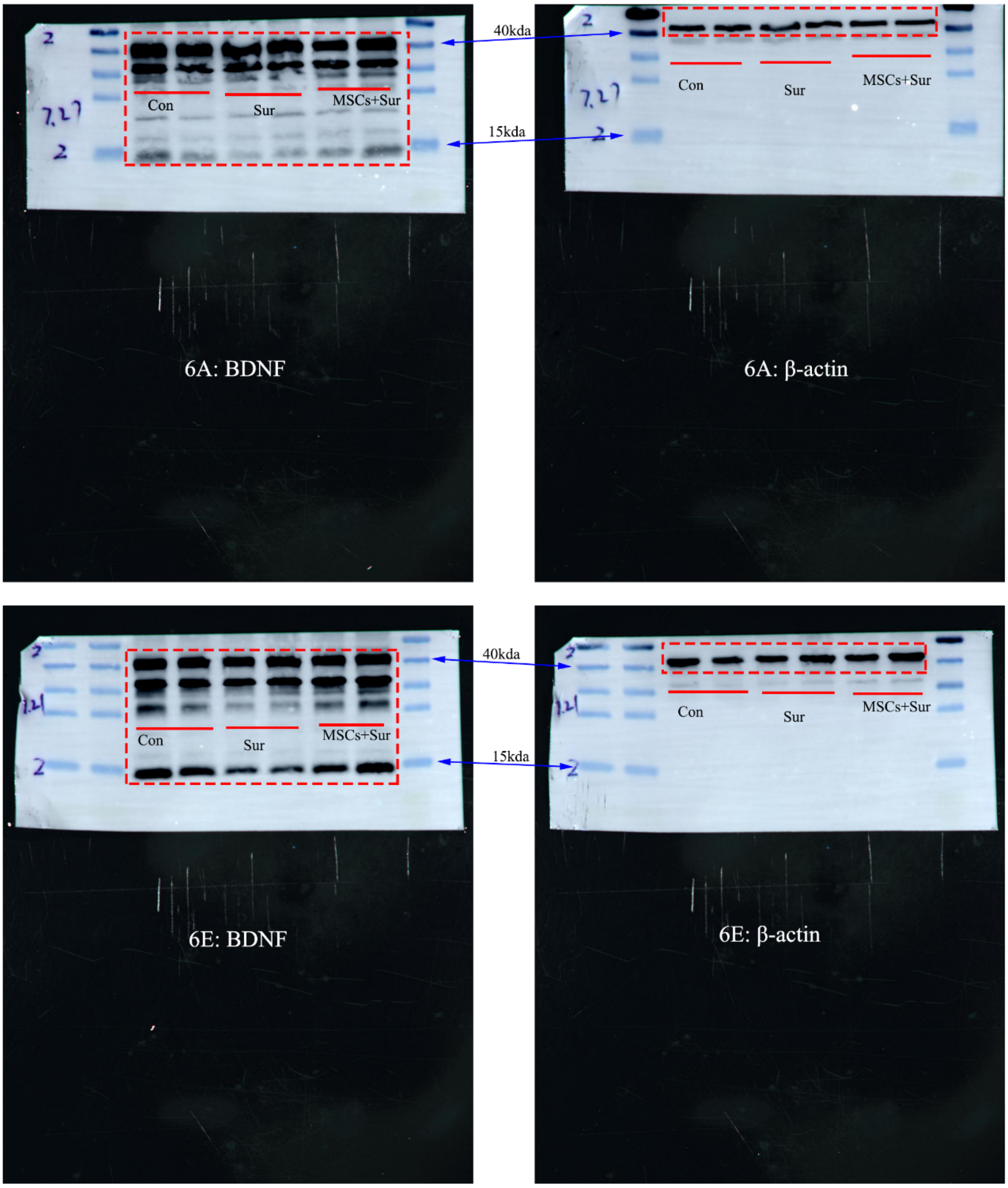

Figure 8A: Original western blot gels of DCX and Sox-2 in the hippocampus. α-Tubulin was included as a loading control.

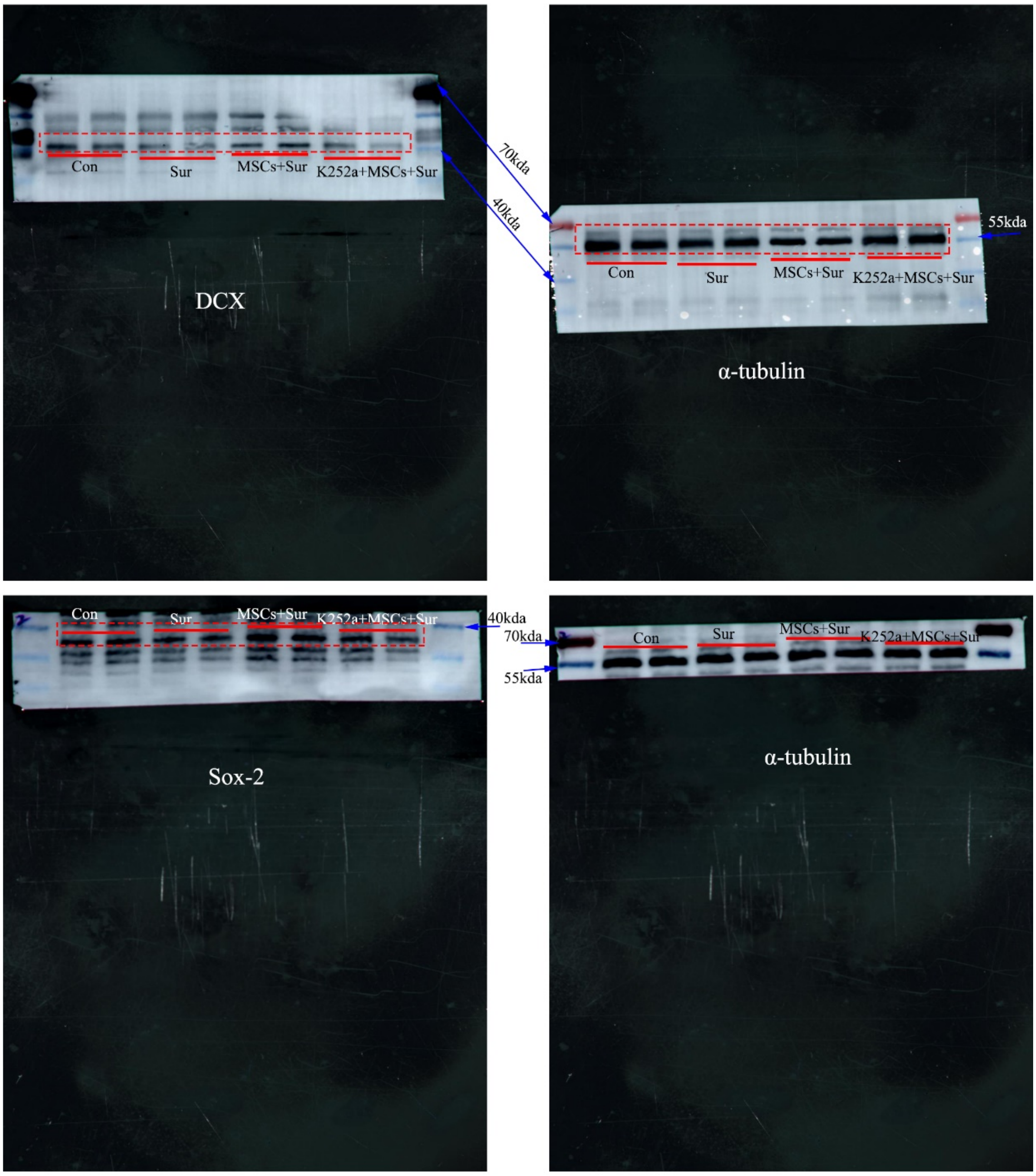

Figure 8G: Original western blot gels of PSD95 in the hippocampus. α-Tubulin was included as a loading control.

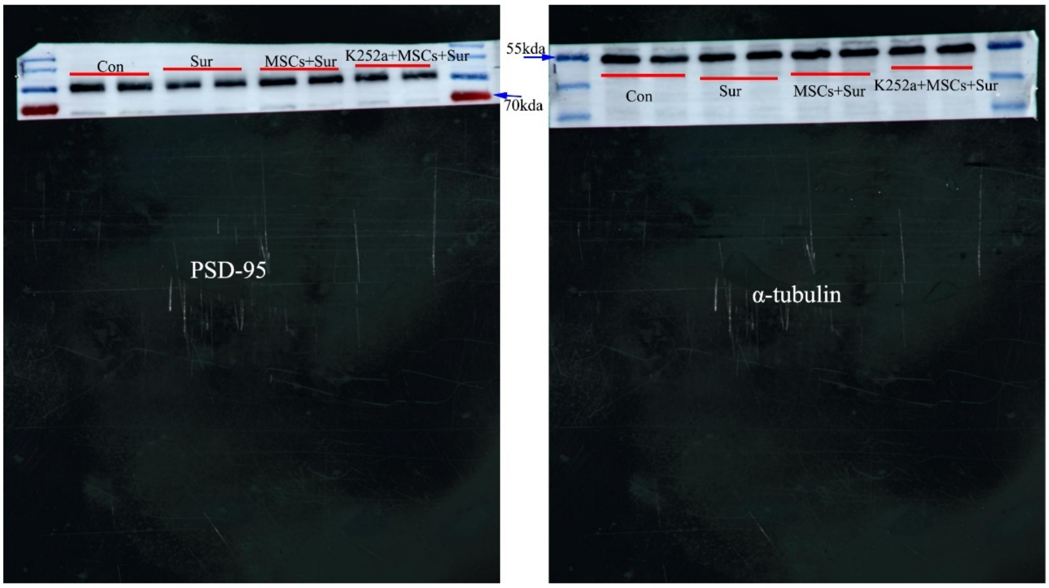

Figure 9A: Original western blot gels of pTrkB/TrkB and pCREB/CREB in the hippocampus. α-Tubulin was included as a loading control.

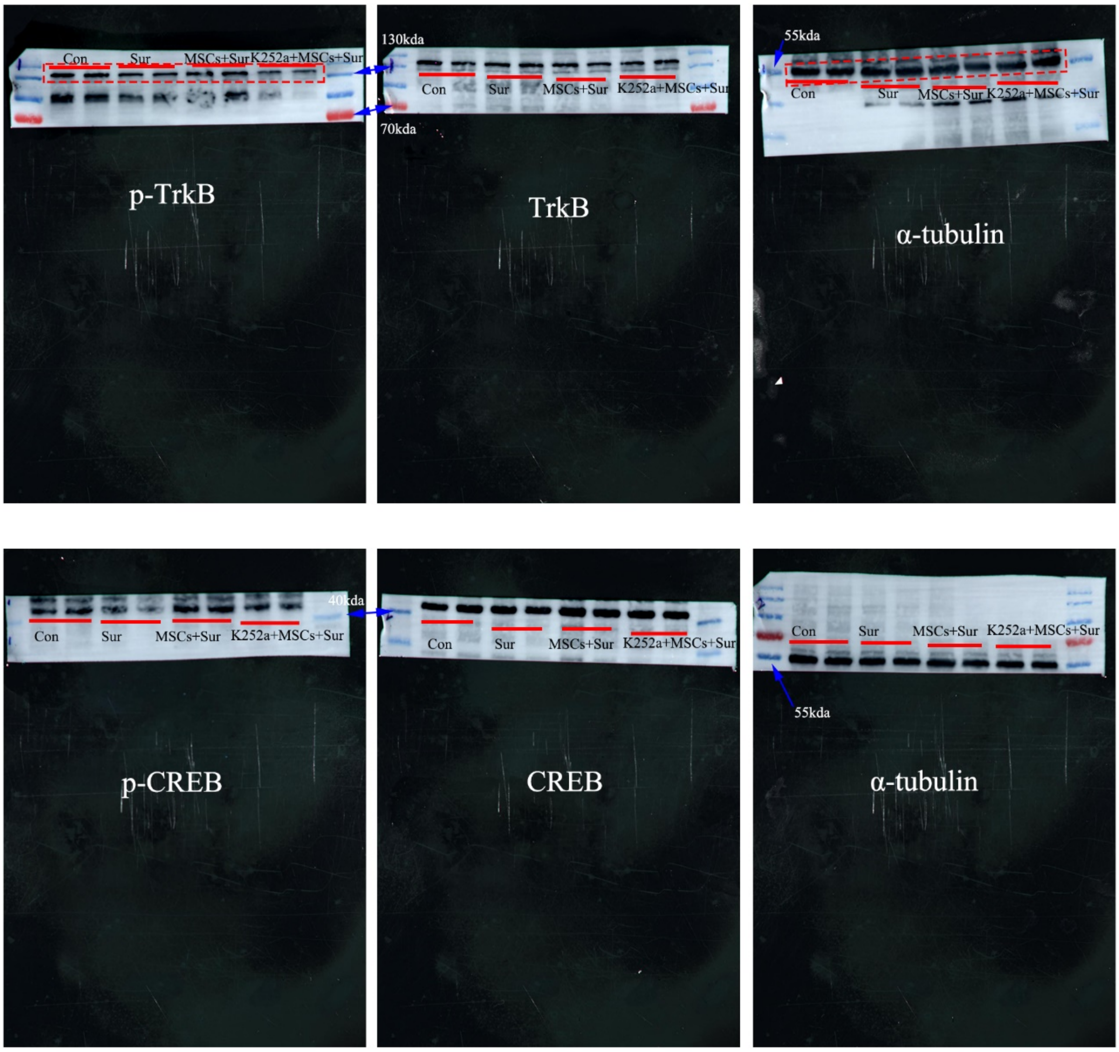

Supplement: Supplementary file 2 — Additional file2. Figure 4A: Original western blot gels of Nestin and Sox-2 in the hippocampus. α-Tubulin was included as a loading control. Figure 5A: Original western blot gels of PSD95 and Syn in the hippocampus. β-actin was included as a loading control. Figure 6A and E: Original western blot gels of BDNF (proBDNF, mature BDNF) in the hippocampus. β-actin was included as a loading control. Figure 8A: Original western blot gels of DCX and Sox-2 in the hippocampus. α-Tubulin was included as a loading control. Figure 8G: Original western blot gels of PSD95 in the hippocampus. α-Tubulin was included as a loading control. Figure 9A: Original western blot gels of pTrkB/TrkB and pCREB/CREB in the hippocampus. α-Tubulin was included as a loading control. [file 13287_2023_3499_MOESM2_ESM.pdf]
